# Characterization of bovine embryos cultured under conditions appropriate for sustaining human naïve pluripotency

**DOI:** 10.1371/journal.pone.0172920

**Published:** 2017-02-27

**Authors:** Bas Brinkhof, Helena T. A. van Tol, Marian J. A. Groot Koerkamp, Richard W. Wubbolts, Henk P. Haagsman, Bernard A. J. Roelen

**Affiliations:** 1 Department of Farm Animal Health, Faculty of Veterinary Medicine, Utrecht University, Utrecht, The Netherlands; 2 Molecular Cancer Research, University Medical Center Utrecht, Utrecht, The Netherlands; 3 Center for Cellular Imaging (CCI), Department of Biochemistry and Cell Biology, Faculty of Veterinary Medicine, Utrecht University, Utrecht, The Netherlands; 4 Department of Infectious Diseases and Immunology, Faculty of Veterinary Medicine, Utrecht University, Utrecht, The Netherlands; University of Texas at Austin Dell Medical School, UNITED STATES

## Abstract

In mammalian preimplantation development, pluripotent cells are set aside from cells that contribute to extra-embryonic tissues. Although the pluripotent cell population of mouse and human embryos can be cultured as embryonic stem cells, little is known about the pathways involved in formation of a bovine pluripotent cell population, nor how to maintain these cells in vitro. The objective of this study was to determine the transcriptomic profile related to bovine pluripotency. Therefore, in vitro derived embryos were cultured in various culture media that recently have been reported capable of maintaining the naïve pluripotent state of human embryonic cells. Gene expression profiles of embryos cultured in these media were compared using microarray analysis and quantitative RT-PCR. Compared to standard culture conditions, embryo culture in ‘naïve’ media reduced mRNA expression levels of the key pluripotency markers *NANOG* and *POU5F1*. A relatively high percentage of genes with differential expression levels were located on the X-chromosome. In addition, reduced *XIST* expression was detected in embryos cultured in naïve media and female embryos contained fewer cells with H3K27me3 foci, indicating a delay in X-chromosome inactivation. Whole embryos cultured in one of the media, 5iLA, could be maintained until 23 days post fertilization. Together these data indicate that ‘naïve’ conditions do not lead to altered expression of known genes involved in pluripotency. Interestingly, X-chromosome inactivation and development of bovine embryos were dependent on the culture conditions.

## Introduction

In mammalian embryos, the first two cell lineages trophectoderm (TE) and inner cell mass (ICM) are established after a series of cleavage divisions. Before implantation, the ICM further delineates into primitive endoderm (PE or hypoblast) and the pluripotent epiblast. It has previously been established that particularly the choice between epiblast and PE is amendable and is influenced by external stimuli [1–3]. Apart from the specification of cell lineages, other crucial events during preimplantation development of placental mammals are activation of the embryonic genome, de- and re-methylation of the genome and, for female embryos, X-chromosome inactivation (XCI) [4–6].

For proper development it is essential that during the peri-implantation stages a pluripotent cell population emerges. Previously we have established that in bovine embryos FGF/ERK-signaling is important to discriminate between pluripotent epiblasts and hypoblasts. Interestingly, cells of the early embryos were found to be very flexible in their differentiation choice, with NANOG-expressing cells being poised for pluripotent epiblasts capable of switching to GATA6-expressing PE [1]. In mice, epiblast cells lose their potential to produce all lineages including the germline, termed naïve pluripotency, shortly after implantation [7].

Recently, a culture medium was reported able to keep human ICM cells in a pluripotent state and that could facilitate the derivation of naïve human pluripotent embryonic stem (ES) cells. Importantly, this medium, termed naïve human stem cell medium (NHSM), containing LIF, growth factors, inhibitors known as 2i [8] and JNK-, p38- and PKC-inhibitors, could also facilitate the conversion of mouse (primed) epiblast stem cells (EpiSCs) to (naïve) ES cells [9] indicating a conserved regulatory network governing naïve pluripotency. Since then, more culture conditions have been described able to capture pluripotency of human embryonic cells (reviewed in [10,11]). Among these are the 3iL medium, containing 2i and an inhibitor of bone morphogenetic protein (BMP) signaling [12], and 5iLA containing 2i, B-RAF, SRC and ROCK-1 inhibitors with LIF and Activin [13]. Since the bovine pluripotency network is poorly understood, culture conditions sustaining human as well as mouse naïve pluripotency might indicate relevant molecules for bovine pluripotency and elucidate how pluripotency is established in the bovine ICM. To identify a transcriptomic profile related to bovine pluripotency, we cultured morula stage bovine embryos in NHSM and compared the ICM mRNA landscape with their counterparts from control in vitro cultured embryos. The expression levels of several genes involved in pluripotency, differentiation and epigenomics were determined in blastocysts cultured in the three different naïve media. Extended embryo culture suggested that these embryos could survive longer in 5iLA than in SOF. Surprisingly, 5iLA bovine embryos could be cultured for over three weeks in vitro with predominantly TE proliferation.

## Materials and methods

### Bovine in vitro embryo production

Oocytes and embryos were cultured in a humidified atmosphere with 5% CO_2_ and 20% O_2_ at 39°C, unless stated otherwise. From bovine ovaries, obtained from a local slaughterhouse, follicles of 3–8 mm in diameter were aspirated to retrieve cumulus oocyte complexes (COCs). COCs in groups of 35–60 per 500 μl M199 (Life Technologies, Bleiswijk, The Netherlands) supplemented with 0.05 IU/ml recombinant hFSH (Organon, Oss, The Netherlands) and 1% (v/v) penicillin-streptomycin (Life Technologies) were matured for 23 hr. Matured COCs were fertilized as described previously [14]. In short, COCs were transferred to fertilization medium (Fert-TALP) supplemented with 10 μg/ml heparin (Sigma-Aldrich, Zwijndrecht, The Netherlands), 20 μM D-penicillamine (Sigma-Aldrich), 10 μM hypotaurine (Sigma-Aldrich), and 1 μM epinephrine (Sigma-Aldrich). Frozen-thawed sperm from a bull with proven fertility was centrifuged over a Percoll-gradient (GE Healthcare Europe GmbH, Eindhoven, The Netherlands) and added to the COCs resulting in a final concentration of 1.0 × 10^6^ spermatozoa/ml. This was considered day 0. After 20 hr, vortexing for 3 min resulted in oocytes freed of cumulus cells, which were placed in synthetic oviductal fluid (SOF) [107.63 mmol/l NaCl (Sigma-Aldrich), 7.16 mmol/l KCl (Sigma-Aldrich), 1.19 mmol/l KH_2_PO_4_ (Sigma-Aldrich), 3.20 mmol/l Sodium DL-lactate (60% syrup; Sigma-Aldrich), 0.74 mmol/l MgSO_4_·7H_2_O (Merck Millipore, Billerica, MA, USA), 25 mmol/l NaHCO_3_ (Sigma-Aldrich), 1.78 mmol/l CaCl_2_·2H_2_O (Sigma-Aldrich), 0.33 mmol/l Sodium pyruvate (Sigma-Aldrich), 2.05 mmol/l LGlutamine (Sigma-Aldrich), 4 mg/ml BSA (Merck Millipore), 10 U/ml penicillin-streptomycin (Life Technologies), 1% MEM NEAA (Sigma-Aldrich), 2% BME Amino Acids (Sigma-Aldrich) and 0.5 μl/ml Phenol Red 0.5% (Sigma-Aldrich) in LAL water (Lonza, Basel, Switzerland)]. After 4 days culturing in a humidified atmosphere with 5% CO_2_ and 7% O_2_ at 39°C, embryos were transferred to fresh SOF, SOF supplemented with KnockOut serum replacement (KOSR; Life Technologies) or to one of the media used for human naïve stem cell generation; NHSM [9], 3iL [12] or 5iLA [13]. Concentrations of media components; recombinant human LIF (Prospec, Rehovot, Israel); recombinant human bFGF (Peprotech; Rocky Hill, NJ, USA); recombinant human TGFβ1 (Peprotech); recombinant human Activin A (Peprotech); PD0325901 (Stemgent, Cambridge, MA, USA); CHIR99021 (Bio-connect, Huissen, The Netherlands), Go6983 (Tocris, Bristol, UK), SP600125 (Tocris); SB203580 (Tocris); WH-4023 (Selleckchem.com; Houston, TX, USA); Dorsomorphin (Sigma-Aldrich); BIO (Sigma-Aldrich); IM12 (Sigma-Aldrich); Y27632 (SigmaAldrich-); SB590885 (Sigma-Aldrich); bovine Insulin (Sigma-Aldrich); NEAA 100x (Sigma-Aldrich); glutamine (Life Technologies); BSA (Life Technologies); N2 supplement (Life Technologies); B27 supplement (Life Technologies); β-mercaptoethanol (Life Technologies); KOSR (Life Technologies); KO DMEM (Life Technologies); TeSR1 (Stem Cell Technologies, Grenoble, France); Neurobasal (Life Technologies); DMEM/F12 (Life Technologies) and penicillin-streptomycin (Life Technologies) are listed in Table 1 (Table 1). For H3K27me3 immunostaining on female blastocysts, matured oocytes were fertilized with sex-sorted (X-chromosome) semen (CRV, Arnhem, The Netherlands) and cultured in SOF, NHSM and 5iLA. At day 8 post fertilization (pf), blastocysts of stage 7 – 9 with quality code 1 and 2 [15] were collected for further processing. To obtain larger proportions of stage 9 (hatched blastocysts) embryos, for ICM isolation and subsequent microarray analysis these embryos were cultured until day 9pf [14,16].

**Table 1:** Composition of culture media used.

SOF	SOF + KOSR	NHSM	3iL	5iLA	Component	Function
*	*	KO-DMEM	TeSR1	1:1 DMEM/F12 & Neurobasal	Basic medium	
-	20%	20%	-	-	KOSR	
-	-	-	-	1%	N2 supplement	
-	-	-	-	2%	B27 supplement	
4 mg/ml	4 mg/ml	-	-	50μg/ml	BSA	
-	-	12.5 μ g/ml	-	-	Rec. Hum. Insulin	
1%	1%	1%	-	1%	NEAA 100x	
2mM	2mM	1mM	-	1mM	glutamine	
2%	2%	0.1mM	-	0.1mM	β-Mercaptoethanol^a^	
0.1%	0.1%	1%	-	1%	pen/strep	
-	-	20 ng/ml	10 ng/ml	20 ng/ml	Rec. Hum. LIF	
-	-	8 ng/ml	-	-	Rec.bFGF	
-	-	1 μ M	1 μ M	1 μ M	PD0325901	MEK-inh
-	-	10 μ M	-	-	SP600125	JNK-inh
-	-	10μM	-	-	SB203580	p38-MAPK-inh
-	-	5 μ M	-	-	Go6983	PKC-inh
-	-	-	-	0.5μM	SB590885	B-Raf-inh
-	-	3μ M	-	-	CHIR99021	GSK3a/b-inh
-	-	-	-	1 μ M	IM-12	GSK3b-inh
-	-	-	2 μ M	-	BIO	GSK3a/b-inh& JAK-inh
-	-	-	2 μ M	-	Dorsomorphin	AMP kinase-inh& BMP-inh
-	-	1 ng/ml	-	-	TGFβ1	TGFβ- SMAD pathway
-	-	-	-	20 ng/ml	Activin A	TGFβ- SMAD pathway
-	-	-	-	1 μ M	WH-4-023	Lck/Src-inh
-	-	-	-	10 μ M	Y27632	ROCK1-inh

* details of SOF can be found in methods section.

^a^ Different suppliers are used for SOF (SigmaAldrich) and NHSM or 5iLA (Life Technologies).

### Generation of bovine parthenogenetically activated embryos

To obtain female embryos solely for the sex ratio determination, parthenogenetically activated embryos were generated. Matured bovine oocytes were stripped from cumulus cells and placed in fertilization medium as described above. The fertilization medium was further supplemented with 5 mM Ionomycin (Sigma-Aldrich) and oocytes were incubated for 5 min. After three times washing in SOF the oocytes were transferred to SOF supplemented with 1.9 mM 6-DMAP (Sigma-Aldrich) and incubated for 3.5 hrs in a humidified atmosphere with 5% CO_2_ and 20% O_2_ at 39°C. Hereafter, the oocytes were washed three times in SOF and cultured in SOF in a humidified atmosphere with 5% CO_2_ and 7% O_2_ at 39°C. At day 5 of culture, cleaved embryos were transferred to fresh SOF and cultured for an additional 4 days when blastocysts were collected for DNA extraction.

### ICM collection and RNA or DNA isolation

ICMs were separated from the TE as described before [14]. In short, blastocysts were placed in wash buffer and sharpened tungsten needles were used to separate the ICM from the TE under a stereo microscope. Isolated ICMs were collected and stored in RLT (Qiagen, Venlo, The Netherlands) in groups of 10–24 at -80°C until RNA isolation. RNA isolation and on column DNA digestion was performed using an RNA micro kit (Qiagen) according to manufacturer’s protocol. A Bioanalyzer 2100 and the RNA 6000 Pico LabChip kit (Agilent Technologies, Amstelveen, The Netherlands) were used for assessment of total RNA quality and quantity per manufacturer’s instructions. RNA was stored at -80°C until further use. DNA from IVF blastocysts and blastocysts from parthenogenetically activated oocytes was extracted using the prepGem kit (ZyGem, Hamilton, New Zealand) according to manufacturer’s instructions and stored at -20°C until further use.

### Microarray analysis

Total RNA samples isolated from dissected ICMs cultured in SOF or NHSM were compared using four dual channel microarrays. On each array a pool of NHSM ICM total RNA was hybridized together with a pool of SOF ICM total RNA. This was performed twice with independent biological replicates. This comparison was repeated with two other biological replicates for each culture condition in a balanced dye-swap. Bovine whole genome gene expression microarrays V2 (Agilent Technologies) representing 43,653 *Bos taurus* 60-mer oligos in a 4x44K layout were used. From each sample 10 ng total RNA was used for complementary DNA (cDNA) synthesis, cRNA double amplification, labelling, quantification, quality control and fragmentation performed on an automated system (Caliper Life Sciences NV/SA, Teralfene, Belgium), all as previously described in detail [17,18]. Microarray hybridization and washing were performed with an HS4800PRO system and Quad-Chambers (Tecan, Mechelen, Belgium) using 500 ng, 1–2% Cy5/Cy3 labelled cRNA per channel as described [17]. Slides were scanned on an Agilent G2565BA scanner at 100% laser power, 30% PMT. After automated data extraction using Imagene 8.0 (BioDiscovery, Hawthorne, CA, USA), Loess normalization was performed [19] on mean spot-intensities. Correction of gene specific dye bias was performed by a within set estimate [20] and data were further analyzed by MAANOVA [21], modelling sample, array and dye effects in a fixed effect analysis. By a permutation F2-test, in which residuals were shuffled 10,000 times globally, p-values were determined. After false discovery rate determination (FDR by Benjamini-Hochberg), gene probes with p<0.05 were considered significantly changed when the difference was at least 1.5-fold. The values from the most 3’ probe were used in case of multiple probes per gene [22]. All microarray gene expression data have been deposited in NCBI’s Gene Expression Omnibus [23] and is accessible through GEO Series accession number GSE69399.

### Complementary DNA generation and (quantitative reverse transcription) PCR

To generate cDNA, RNA was converted using the iScript cDNA Synthesis Kit (Bio-Rad) according to manufacturer’s protocol. For primers (S1 Table) to be designed a primer3 based platform (Primer-Blast; http://www.ncbi.nlm.nih.gov/tools/primer-blast) was used [24] with specific *Bos taurus* mRNA templates (Genbank; http://www.ncbi.nlm.nih.gov/nucleotide). Four-times dilution series of cDNA from blastocysts were used for confirmation of primer pair specificity and establishing annealing temperatures (Ta) by performing a temperature gradient ranging from 57–65°C. DNA or cDNA for all PCR or quantitative reverse transcription PCR (qRT-PCR) reactions respectively was mixed with 25 μl iQ SYBR Green supermix (Bio-Rad) with a final primer concentration of 400 nM in 25 μl reaction volume and measured on a CFX detection system (Bio-Rad) according to manufacturer’s protocol. Reactions started with 5 min at 95°C for enzyme activation followed by 40 cycles of first a denaturing step at 95°C for 10 s followed by an annealing step at appropriate Ta (S1 Table) for 10 s and finally a 30 s elongation step at 72°C. The reaction continued with the generation of a dissociation curve by increasing the temperature by 1°C for 15 s each step from 60°C to 98°C. Relative gene expression levels were calculated using the comparative C_T_ method (described in Applied Biosystems User Bulletin No. 2 [P/N 4303859]). Normalization for qRT-PCR- data was performed using reference genes and for relative DNA copy numbers two genes located on the autosomes were used (S1 Table). All calculations were performed in CFX manager software (Bio-Rad) except statistical analysis which was a student t-test after determination of equality of variances in Excel.

### Bovine ICM and embryo culture in 5iLA

Laminin-521 (Biolamina, Sundbyberg, Sweden) / E-cadherin (R&D Systems) coating was performed as previously described [25]. Tissue culture plates were coated at a concentration of 13.5μg/ml Laminin-521 and 1.5μg/ml E-cadherin in DPBS containing Ca^2+^ and Mg^2+^ (Life Technologies) for 2h at 37°C. Next the plates were washed twice with DPBS containing Ca^2+^ and Mg^2+^ (Life Technologies) and used immediately.

Coating with Cell-Tak (Corning, Bedford, MA, USA) adhesive was performed as recommended by supplier. Stock solution was diluted in NaHCO_3_, pH 8.0 and 1N NaOH to obtain a concentration of 3.5 μg Cell-Tak/cm^2^. Incubation was performed at room temperature for 1h and then plates were washed twice with DPBS containing Ca^2+^ and Mg^2+^ (Life Technologies). Plates were used immediately or air-dried and stored at 4°C until use. The ICM was dissected from day 8 embryos cultured in either SOF, 3iL, 5iLA or NHSM as described above and placed in any one of the coated wells. Passaging of colonies was done mechanically using fine glass Pasteur’s pipettes each 7th day. Medium was replaced every 2–3 days.

Intact embryos in 5iLA medium were cultured until day 23pf in uncoated plates. From day 12pf, embryos were transferred to fresh medium every other day and from day 17pf, half of the culture medium was replaced by fresh medium every day.

### Immunostaining

Collected blastocysts were fixed in 4% paraformaldehyde (PFA) for 15 min and stored in 1% PFA at 4°C until further use. Subsequent steps were performed at room temperature unless stated otherwise. Blastocysts were washed briefly in PBS containing 0.1% Triton X100 (Sigma Aldrich) and 10% FCS (PBST) and permeabilized in PBS + 0.5% Triton X100 + 10% FCS for 30 min. For detection of GATA6 and NANOG, blastocysts were blocked for a-specific binding by 1 hour incubation in PBST and subsequently incubated overnight with the primary antibodies rabbit anti-GATA6 (Santa Cruz, Dallas TX, USA; sc-9055; 1:100) and mouse anti-NANOG (eBiosciences, San Diego CA, USA; 14-5768-82; 1:250) at 4°C. Secondary antibody incubation was performed for 1 hour with secondary antibodies goat anti-mouse Alexa 647 and goat anti-rabbit Alexa 488 (both Life Technologies; 1:200) followed by 5 min incubation with DAPI (0.1 μg/ml) (Sigma-Aldrich). To detect tri-methylation on Lys27 of histone H3 a rabbit monoclonal antibody against H3K27me3 (Cell Signaling Technologies; #9733; 1:1000) was used at 4°C during overnight incubation and subsequently incubated for 1 hour in goat anti-rabbit Alexa 568 antibody (Life Technologies; 1:200) and 5 min in TOPRO3 (Life technologies; T3605; 10μM) fur nuclear staining. Nuclear together with lipid droplet staining was performed for 15 min with DAPI (0.1 μg/ml) and LD540 (0.05 μg/ml), respectively. Lipid droplet staining dye LD540 was kindly donated by Dr. C. Thiele, Bonn, Germany via Maidina Tuohetahuntila, Utrecht, The Netherlands. Embryos were mounted in Vectashield (Brunschwig Chemie, Amsterdam, The Netherlands) in Grace Bio-Labs SecureSeal imaging spacers (Sigma-Aldrich) and overlaid with optical coverslips (Knittel Gläser, Braunschweig, Germany). Embryos were imaged with an inverted semi-automated confocal microscope (SPE-II–DMI4000; Leica, Son, The Netherlands) and analyzed using Fiji software [26]. Image acquisition of the H3K27me3 and TOPRO3 signal was performed on a Nikon A1R+ confocal scanning microscope. Stacks of images were acquired by carefully focusing the middle of each embryo and a range of 75 μm above and under this position was covered with step sizes of 2 μm. Processing of these images was done using NIS-Elements (Nikon, Amsterdam, The Netherlands) and IMARIS 8.1 (Bitplane AG, Zurich, Switzerland) to render the 3D projections and facilitate the classification of H3K27me3 staining using the object viewer of the Vantage module. The individual cells per embryo were analyzed by 2 independent researchers (double-blinded) for presence of a H3K27me3 positive patch.

## Results

### Culture of embryos in naïve media

**Fig 1:**
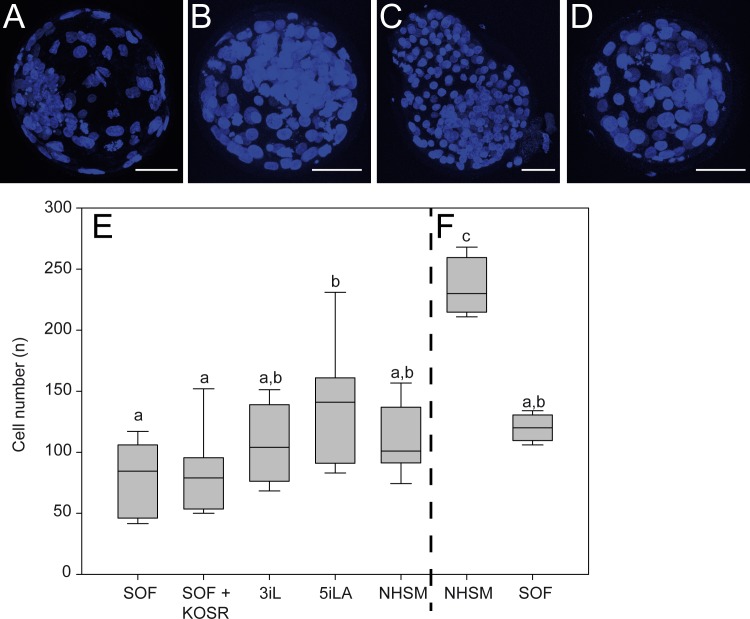
Bovine in vitro embryo culture in human ‘naïve’ media. In embryos cultured in SOF (A), 3iL (B), 5iLA (C) and NHSM (D) cells were identified by nuclear DAPI staining (blue) and the average cell number (±SD) was determined in day 8pf (E) and day 9pf embryos (F). Scale bar represents 50μm. Significant differences (p<0.05) are indicated by different letters.

Bovine in vitro produced embryos were cultured in standard SOF, a basic medium based on MEM [2], for 4 days. To investigate the importance of the culture medium on the formation of the pluripotent cell population in blastocysts, morula stage embryos were further cultured in three different ‘naïve’ media: NHSM [9], 3iL [12] and 5iLA [13] and compared with standard SOF-cultured embryos (Table 1 and Fig 1A–1D). These naïve media all contained LIF and compounds inhibiting GSK3β and MAPK known as 2i [8]. The 3iL medium was further supplemented with a BMP-inhibitor, known to reduce the BMP induced down-regulation of *NANOG* in human ES cells [27]. NHSM and 5iLA contained members of the TGFβ –SMAD pathway (TGFβ1 and Activin A respectively) enhancing *NANOG* transcription in human ES cells [28]. NHSM furthermore contained bFGF and inhibitors of JNK, PKC, and p38 whereas 5iLA was further supplemented with a B-RAF-, an SRC-, and a ROCK1-inhibitor.

**Fig 2:**
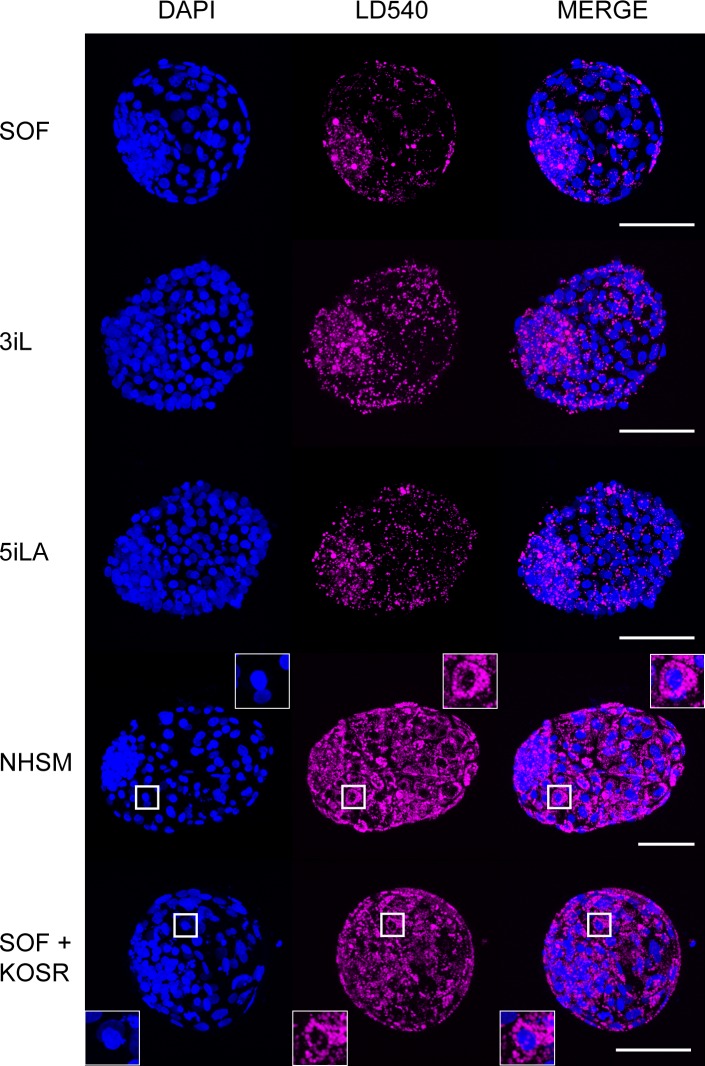
Lipids in bovine embryos. Representative pictures of embryos cultured in SOF, 3iL, 5iLA, NHSM, and SOF supplemented with KOSR stained by LD540 (pink) for lipid droplets and counterstained with DAPI (blue)are shown. Scale bars represent 100mm. Inserts show a higher magnification of trophectoderm cells with perinuclear lipid droplets.

The percentage of embryos that had developed to the blastocyst stage at day 8pf was similar in each group with no difference in the percentage of embryos hatched from their zonae pellucidae. Cell number has been reported as a measure for embryo quality [29] and therefore cell numbers of day 8pf blastocysts (n = 7–10) were determined (Fig 1E and for individual scores S1A Fig). SOF-cultured embryos at day 8pf were composed of the smallest number of cells (81 ± 29) whereas day 8pf embryos cultured in 5iLA contained the largest number of cells (141 ± 49). Furthermore, day 9pf NHSM blastocysts (n = 4) displayed an almost 2-fold increase in cell number compared to day 9pf SOF-cultured embryos (n = 4) (1.96-fold; p = 0.0007, Fig 1F and for individual scores S1B Fig). Although ICM cell percentage between embryos was variable, the expression of GATA6 and NANOG in the ICM was not significantly altered in the different culture media (S1C and S1D Fig). Nevertheless, larger embryos (>100 cells), cultured in 5iLA contained a higher percentage of NANOG expressing cells in the ICM (S1C Fig). The NHSM-cultured blastocysts appeared darker than the embryos in the other groups and hatched embryos were more buoyant, possibly because of lipid accumulation. Indeed, the NHSM-cultured embryos contained numerous perinuclear lipid droplets in both TE cells and ICM cells whereas lipid droplets in embryos from SOF, 3iL or 5iLA were predominantly present in the ICM and only a few in the TE cells (Fig 2). Since serum enhances lipid droplet formation [30], the presence of serum replacement in NHSM may be the cause of the lipid droplet distribution. Indeed, blastocysts cultured in SOF with 20% serum replacement (KOSR) (Table 1) showed similar lipid droplet distribution as in NHSM blastocysts (Fig 2).

### Gene expression in the ICM of NHSM-cultured embryos

We next used microarray analysis to examine gene expression patterns of ICM cells from blastocysts cultured in NHSM in comparison to those cultured in SOF. Embryos were cultured until day 9pf after which the ICMs were manually isolated and subjected to RNA extraction. Because for the manual isolation, the ICMs were not entirely pure and previously we had established that the ICMs contained approximately 20% cells with TE characteristics [14]. Only those samples with good RNA quality, indicated by RNA integrity number (RIN) values above 8, were used. From each culture condition four separate pools of ICMs were generated and compared as such that one NHSM pool was hybridized on an array together with one SOF pool labeled with either Cy5 or Cy3. This resulted in a total of four microarrays analyzed.

**Fig 3:**
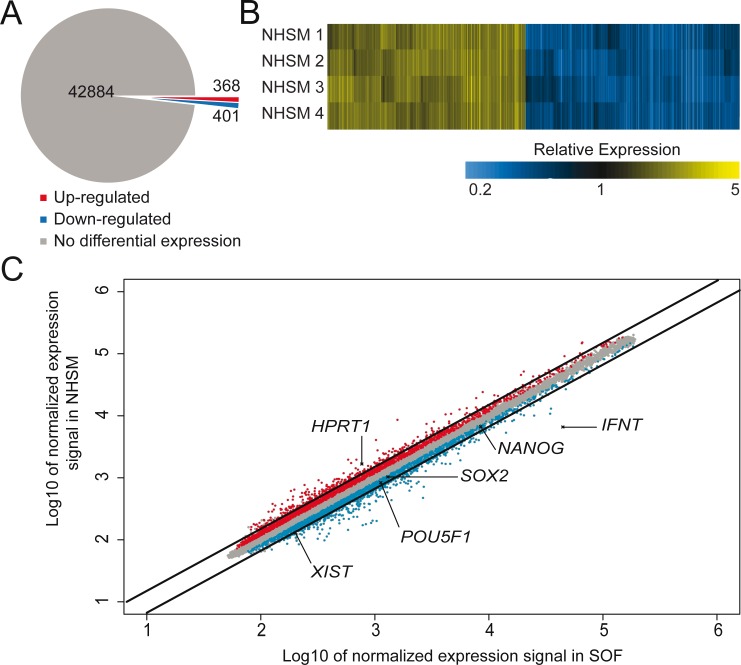
Microarray data. Pie diagram (A) showing the numbers of genes examined by microarray analysis. Number of genes up regulated in NHSM medium is indicated in red and down regulated gene number in blue. Heatmap comparing four different comparisons between NHSM and SOF ICMs (B). These results were obtained from four separate microarrays showing a similar expression in NHSM-cultured embryos relative to SOF-cultured embryo expression. Scatter plot indicating differences in gene expression between Inner Cell Mass cells from NHSM-cultured and SOF-cultured embryos (C). Probes expressed at significantly higher levels in NHSM ICMs (red), in SOF ICMs (blue) or with no significantly different expression levels (grey) are depicted in this scatter plot. Diagonal lines indicate the 1.5-fold cut off used for analysis. Several genes discussed in the manuscript are indicated.

**Fig 4:**
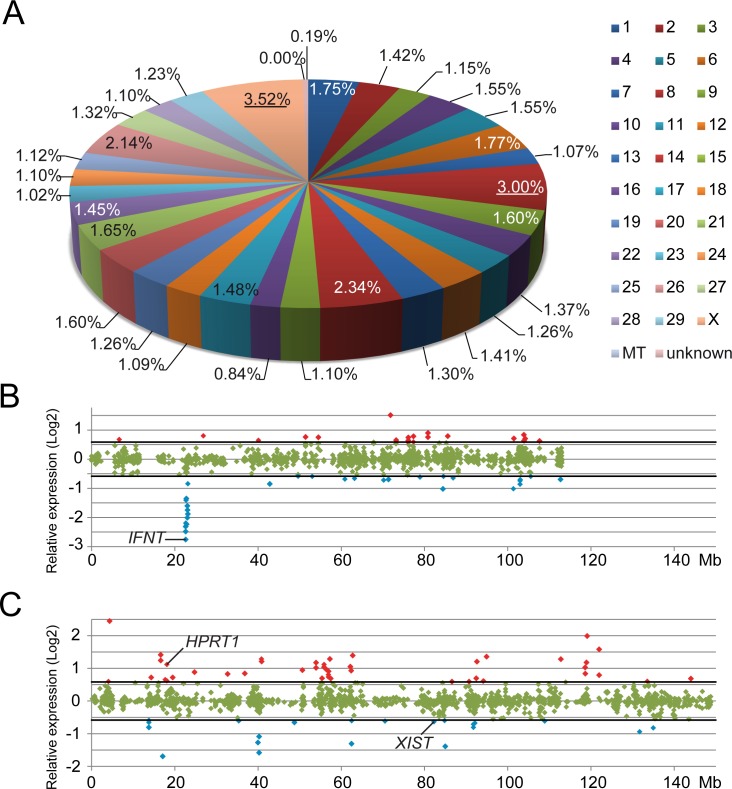
Chromosome distribution. Proportional distribution of differentially expressed probes per chromosome, mitochondrion (MT) or of unknown location (A). Chromosome 8 (red; 3.00%) and the X chromosome (pink; 3.52%) were overrepresented (as indicated with the underlined percentages) by differentially expressed probes. The relative expression (Log2) of probes with higher (cut off 1.5-fold change indicated with thick black lines) expression levels in NHSM (red) or in SOF (blue) developed ICMs are plotted with respect to the location on chromosome 8 (B) and the X-chromosome (C).

**Fig 5:**
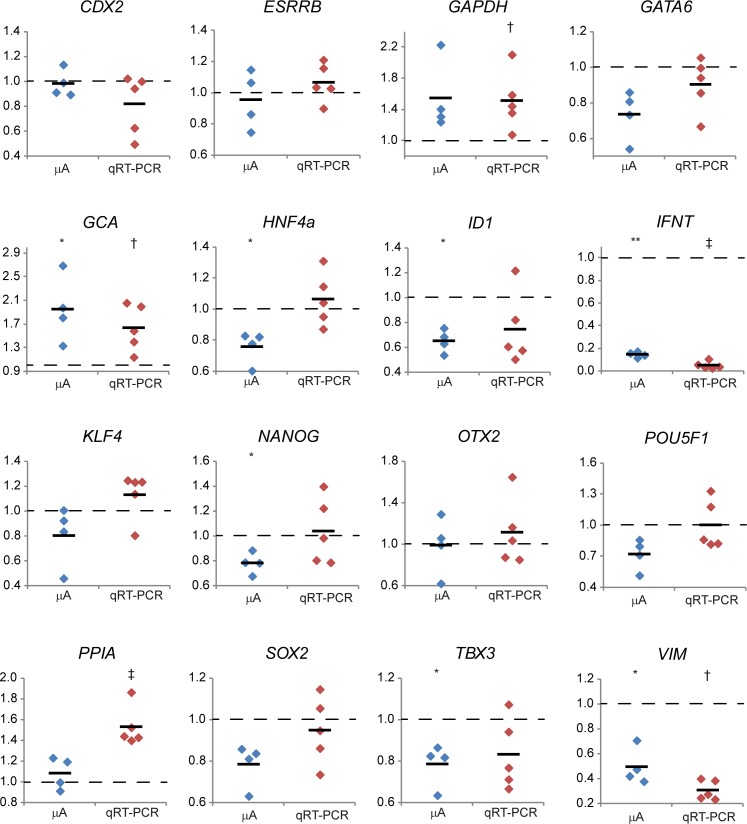
Relative gene expression levels in ICMs from day 9pf embryos. The expression of selected genes in SOF-cultured ICMs is set at 1 (dashed line) to show the relative gene expression (y-axis) in NHSM-cultured ICMs after microarray analysis (mA; blue) and qRT-PCR (red). Levels of significantly different expression (p<0.05 or p<0.01, respectively) are indicated by single or double asterisks or single or double dagger in microarray or qRT-PCR, respectively.

Microarray expression profiles revealed 769 probes to be differentially expressed (cut-off 1.5-fold; p<0.05, FDR corrected) between NHSM- and SOF-cultured ICMs, from a total of 43,653 probes (Fig 3A). Comparing the four array results by generating a heatmap showed similar expression profiles indicating the reproducibility (Fig 3B). Expression levels of 368 probes were higher in ICMs from NHSM-cultured embryos whereas 401 were more abundantly expressed in ICMs from SOF-cultured embryos (Fig 3C). According to an Ensembl gene count (http://www.ensembl.org/biomart/martview/) the *Bos taurus* genome contains 24,616 genes. Approximately 80% of these genes were represented on the microarray, equally distributed over the chromosomes. Also, the probes per gene ratios (≈ 2.2) are similar for all chromosomes. The distribution of the differentially expressed probes on the *Bos taurus* genome was comparable for all chromosomes (average = 1.52% ± 0.58) apart from chromosome 8 (3.0%) and the X chromosome (3.5%) that had a higher percentage of differentially expressed genes (Fig 4A). On chromosome 8 the largest expression difference was detected in a confined region between 22.6 Mb and 23.2 Mb containing interferon (IFN) coding genes including *IFNW*, *IFNA* and *IFNT* (Fig 4B). The X-chromosome exhibited a more diverse pattern of differentially expressed probes of which 44 were >1.5-fold up-regulated and 22 down-regulated in NHSM (Fig 4C). Since several genes were represented on the microarray by more than one probe and for consistency, we used the probes representing the most 3-primed ends of the genes. Accordingly, a remaining 641 genes were differentially expressed (S2 Table). A list of the 25 most differentially expressed genes also indicates an overrepresentation of genes located on the chromosomes X and 8 (Table 2). A selection of 16 genes known to be involved in embryonic development and pluripotency were assessed for their expression levels by qRT-PCR, and indeed gene expression levels in SOF- and NHSM-cultured ICMs were similar to the microarray data for these 16 genes (Fig 5).

**Table 2:** List of most differentially expressed genes.

Gene Symbol (NCBI)	Description	Chr.	NHSM vs SOF (FC)
IFN-tau-c1	Interferon-tau 3g precursor	8	-6.75
CT47B1	Cancer/testis antigen family 147, member B1	X	5.45
DUSP4	Bos taurus dual specificity phosphatase 4 (DUSP4).	27	-5.15
LOC781778	Uncharacterized protein	8	-4.71
ENSBTAG00000046119	Uncharacterized protein	8	-4.67
LOC100847720	Uncharacterized protein	8	-4.56
ENSBTAG00000034320	Novel pseudogene	8	-4.03
LOC101905249	Melanoma-associated antigen B1-like	X	3.97
ABCC2	Uncharacterized protein	26	-3.76
LOC523509	Uncharacterized protein	8	-3.69
DAZL	Deleted in azoospermia-like	1	3.36
LOC618985	Uncharacterized protein	8	-3.35
LOC618806	Melanoma-associated antigen 10-like	10	3.34
GPC4	Glypican-4 precursor	X	-3.21
ENSBTAG00000045727	Novel pseudogene	14	-3.19
ACTA1	Actin. alpha skeletal muscle	28	3.17
PDYN	Proenkephalin-B preproprotein	13	-3.13
LOC100336885	Uncharacterized protein	8	-3.03
TKTL1	Transketolase-like protein 1	X	-3.00
LOC520085	Bos taurus similar to melanoma antigen (LOC520085).	X	2.99
QPRT	Nicotinate-nucleotide pyrophosphorylase	25	-2.99
PLAU	Urokinase-type plasminogen activator	28	-2.94
SPRY4	Sprouty homolog 4 (Drosophila)	7	-2.93
SLC13A4	Solute carrier family 13 member 4	4	-2.87
STC1	Stanniocalcin-1	8	2.85

The 25 genes with the largest expression difference after microarray analysis between NHSM- and SOF-cultured ICMs are presented. Negative values indicate fold change (FC) down-regulated in NHSM and positive values are up-regulated.

Since human embryonic cells cultured with NHSM acquire a more naïve signature, we were surprised that in the bovine ICMs the expression of core pluripotency genes *NANOG*, *SOX2* and *POU5F1* was at similar levels (*SOX2*, *POU5F1*) or down-regulated (*NANOG*) in NHSM, although this down-regulation was below the 1.5-fold cut-off (Fig 3C) and could not be confirmed by qRT-PCR (Fig 5). Genes of which, at least in the mouse, the products direct OCT4 to enhancer sites related to a primed state [31] were inconclusively expressed. Whereas *OTX2* (Fig 5) and *ZIC3* (S2 Table) showed no significant differential expression (-1.01-fold, p = 0.987 and 1.20-fold, p = 0.064 respectively), *ZIC2* exhibited marked down-regulation (2.01-fold, p<0.001) in NHSM (S2 Table). Expression of other transcription factor genes associated with self-renewal and pluripotency was either unchanged (FC<1.1-fold; *DPPA2*, *TFCP2L1*, *NR5A2* and *ESRRB*), not significantly changed (p>0.05; *KLF2* and *KLF4*) or moderately down-regulated (1.5>FC>1.25, p<0.05; *DPPA3*, *KLF5* and *TBX3*) in NHSM (S2 Table). Results from qRT-PCR data showed similar expression dynamics although most expression levels were not significantly different between NHSM and SOF (Fig 5). Taken together, the similarity in expression of pluripotency genes suggests a normal developmental pattern of bovine embryos in NHSM medium.

### Quantitative PCR analysis of bovine embryos cultured under human naïve conditions

Next, specific gene expression levels were determined in embryos cultured in the three different ‘naïve’ media and compared with embryos cultured in SOF. At day 8pf, 15–26 embryos per culture group were pooled and RNA was extracted resulting in comparable amounts per embryo. Expression levels of genes involved in embryonic cell type specification, pluripotent state, epigenetic regulation and differentiation were determined in whole embryos by qRT-PCR (S2 Fig). Expression of PE-markers *FGFR2* and *GATA6* was found to be down-regulated in all ‘naïve’ media compared to SOF embryos. Surprisingly, core pluripotency markers *NANOG* and *SOX2* were not differentially expressed whereas *POU5F1* was down-regulated when cultured in the ‘naïve’ media.

**Fig 6:**
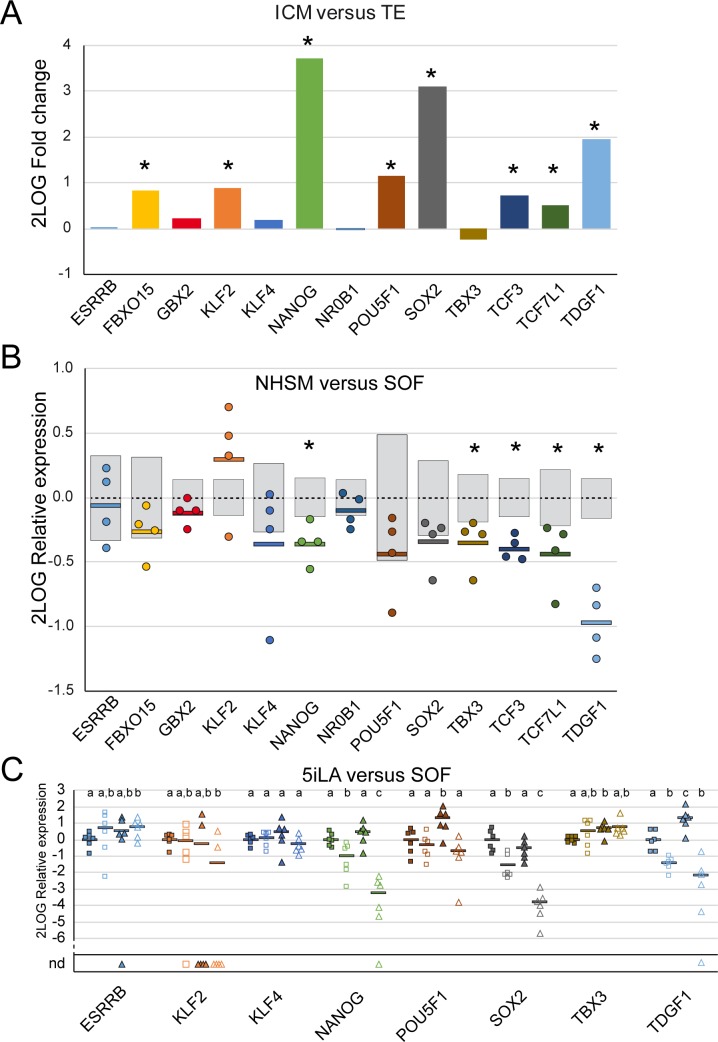
Expression differences of pluripotency related genes in the ICM and TE. Microarray data (GEO accession no. GSE63054; [14]) were analyzed for the differential expression of a selection of pluripotency related genes comparing dissected ICM and TE from bovine day 9pf embryos cultured in SOF (A). 2Log TE expression was set at 0 and significant differences indicated by an asterisk (*). The same genes were assessed in the microarray performed for this study (B). 2Log average ICM expression levels (dashed lines) from SOF-cultured embryos was set at 0 with grey bars indicating SD. Relative 2Log expression levels in ICMs from NHSM-cultured embryos are depicted by dots and averages indicated by a horizontal bar. Significant average expression differences are indicated by an asterisk (*). Quantitative RT-PCR was performed to determine expression of 8 selected genes in the ICM (closed symbols) and TE (open symbols) of 5iLA- (squares) and SOF-cultured (triangles) day 9pf embryos (each 6 groups of 10 ICMs or TEs). 2Log average expression in the 5iLA-cultured ICM was set at 0 per gene. ICM or TE samples without detectable gene expression are indicated as nd. 2Log average gene expression is indicated by a horizontal bar and significant differences indicated by different letters.

Previously, we established a transcriptomic landscape of the ICM and TE in SOF-cultured day 9pf bovine embryos [14]. From these data a selection of genes known to be involved in (mouse) pluripotency was assessed for their differential expression levels in bovine ICM and TE (Fig 6A). Interestingly, *ESRRB*, *KLF4* and *TBX3*, known pluripotency related genes in the mouse and primate [7], were not differentially expressed in the ICM and TE of bovine embryos. Expression levels of the same panel of genes were compared in the ICM of NHSM- and SOF-cultured bovine embryos (Fig 6B). NHSM-culture caused a 1.28-fold (p = 0.018) down-regulation of *NANOG* expression without a significant alteration of *POU5F1* or *SOX2* expression levels. Expression levels of *TBX3*, *TDGF1*, *TCF3* and *TCF7L1* were also reduced in ICMs isolated from NHSM-cultured embryos.

We next compared the expression levels of these genes in the ICM and TE of bovine embryos cultured in 5iLA or SOF (Fig 6C). The expression differences between ICM and TE from SOF-cultured embryos determined by qRT-PCR (Fig 6C) were comparable with the differential expressions obtained from the microarray data (Fig 6A). Like NHSM, 5iLA did not alter the expression levels of *ESRRB*, *KLF2*, *KLF4* and *SOX2* in the ICM. Although *NANOG* expression was also reduced in 5iLA (1.39-fold) this was not significant (p = 0.194). Expression of *POU5F1* was 2.46-fold (p = 0.028) down-regulated in the ICM from 5iLA-cultured embryos. Embryo culture in 5iLA also resulted in reduced expression levels of *TBX3* and *TDGF1*. In TE, 5iLA did not alter expression levels of most pluripotency related genes. Nevertheless, expression levels of *NANOG* and *SOX2* were dramatically increased in the TE part of the 5iLA embryos compared to expression levels of these genes in the TE from SOF-cultured embryos.

Taken together, despite some differences, culture of bovine embryos in 5iLA and NHSM does not seem to enhance the pluripotency network in bovine ICMs.

### X—Chromosome inactivation

**Fig 7:**
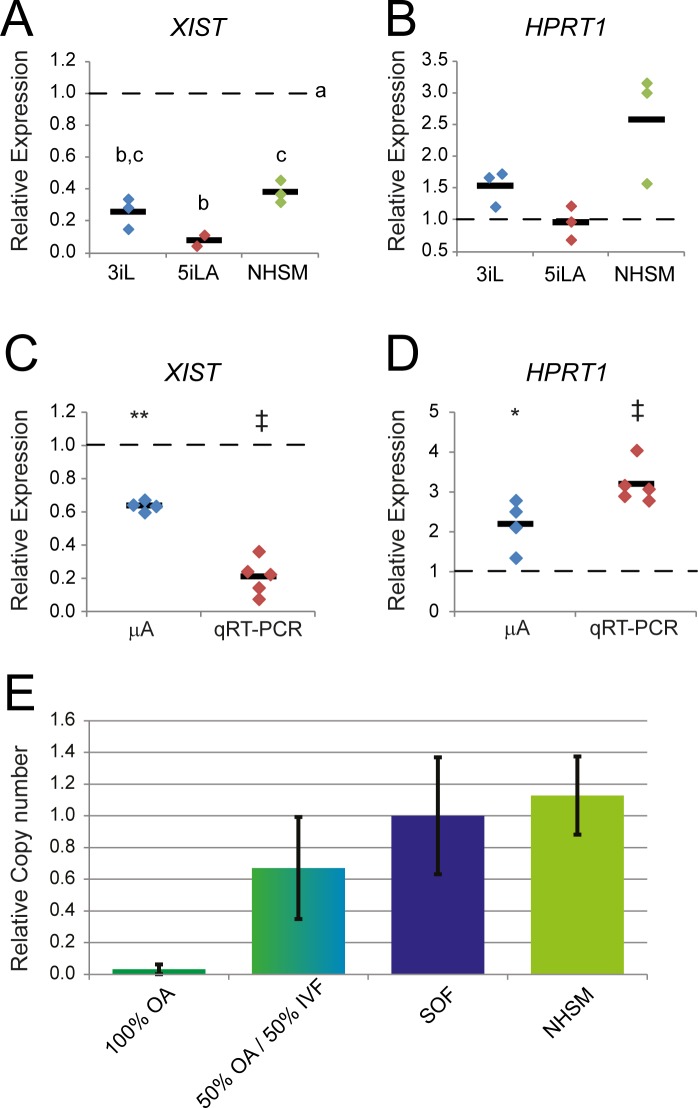
XCI in bovine embryos. Relative expression levels of *XIST* (A) and *HPRT1* (B) were determined by qRT-PCR in day 8pf embryos cultured in 3iL (blue), 5iLA (red) and NHSM (green) and compared with SOF day 8pf embryonic expression levels set at 1 (dashed line). Relative expression levels of *XIST* (C) and *HPRT1* (D) in ICMs dissected from day 9pf embryos cultured in NHSM were obtained by microarray (mA; blue) and qRT-PCR (red) and compared with their SOF counterparts set at 1 (dashed line). Relative copy numbers (mean ± SD) of genomic SRY (E) was determined in parthenogenetically activated oocytes (OA), a combination of OA and IVF produced blastocysts cultured in SOF (green/blue), IVF SOF-cultured blastocysts alone (purple; set at 1), and cultured in NHSM (green) by PCR.

During mouse embryogenesis, the inactivated paternal X-chromosome is reactivated in the epiblast cells of a preimplantation blastocyst resulting in a pluripotent cell population with two active X-chromosomes. Soon after implantation random X-chromosome- inactivation (XCI) is initiated as a result of expression of the non-coding RNA *Xist*. Interestingly, all naïve culture media caused a reduction in *XIST* expression of at least 2.5-fold (Fig 7A) in bovine day 8pf embryos. *HPRT1*, located on the X-chromosome, is one of the genes of which the expression is down-regulated after XCI and expression levels of this gene are frequently used as a marker for XCI [32]. Despite decreased *XIST* expression however, the expression levels of *HPRT1* in day 8pf whole embryos cultured under the ‘naïve’ conditions did not significantly differ from those of control embryos (Fig 7B). In whole female embryos, a decreased expression of *HPRT1* in the TE cells, resulting from paternal XCI as has been observed in mouse embryos ([33] for review), might obscure the up-regulation in the ICM. Indeed, *XIST* expression was significantly reduced (Fig 7C) and the expression of *HPRT1* was significantly up-regulated (Fig 7D) in the dissected ICMs from day 9pf NHSM embryos compared to the expression in ICMs from SOF-cultured day 9pf embryos. With embryos of mixed sex, a reduction of *XIST* expression could be due to a higher percentage of male embryos that are expected to have lower *XIST* levels. Therefore, we examined whether culture in NHSM skewed the sex ratio with a higher percentage of male embryos reaching the blastocyst stage. In embryos from parthenogenetically activated oocytes, the Y-chromosome specific *SRY* gene was not present, and the genomic *SRY* copy numbers increased when we mixed these samples with those from mixed sex IVF embryos (Fig 7E). In SOF and NHSM blastocysts the relative copy numbers of *SRY* were similar indicating that there was no difference in the sex ratio of embryos cultured in SOF and NHSM (Fig 7E).

**Fig 8:**
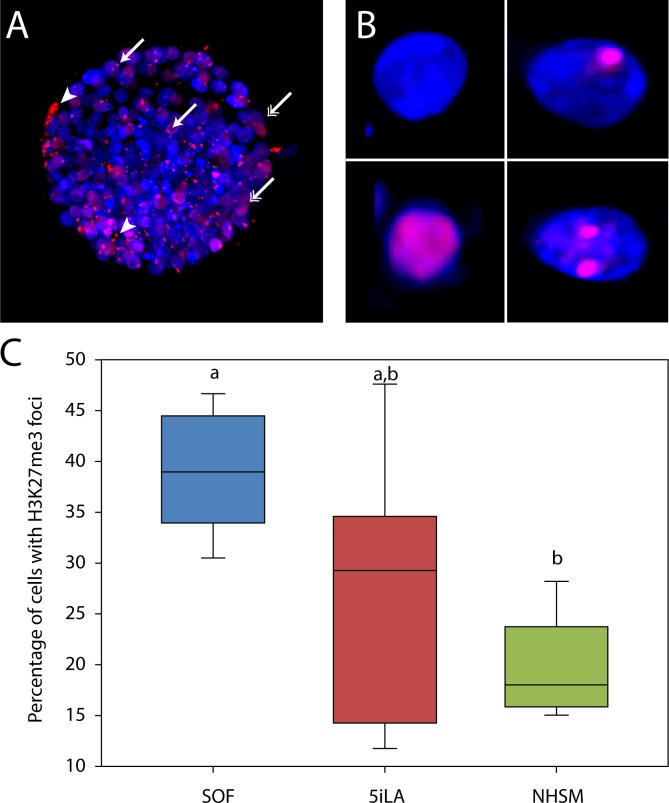
H3K27me3 in bovine embryos. Bovine day 8pf embryos cultured in SOF, 5iLA or NHSM were stained for H3K27me3 (red; representative picture in A; a-specific staining indicated by arrow heads). Nuclei (blue) without foci (A; double headed arrows and B; upper left and lower left) were scored as active X-chromosome, whereas nuclei containing ≥1 foci (A; single headed arrows and B; upper right and lower right) were scored as an inactive X-chromosome (Xi). The average percentage of cells (± SD) with H3K27me3 foci per embryo from 5 SOF embryos (blue), 7 5iLA embryos (red) and 5 NHSM embryos (green) was calculated (C). Different letters indicate significant differences (p<0.05).

In the mouse, *Xist* expression recruits the polycomb-represssive complex 2 (PRC2), which leads to tri-methylation of Lysine 27 on histone 3 (H3K27me3). H3K27me3 is associated with gene silencing and together with *Xist* coating essential to establish and maintain XCI [34]. Cells in female day 8pf embryos were analyzed for their H3K27me3 staining (Fig 8A and 8B, S1 File and S2 File). Different variants of H3K27me3 labelling were observed: no labelling, a single positive nuclear patch, diffuse nuclear distribution or multiple nuclear foci (Fig 8B). Only those cells with a clear isolated nuclear patch were scored as having an inactivated X-chromosome. In SOF-cultured embryos, foci of H3K27me3 were observed in ≈ 40% of the cells. Compared to the SOF-cultured embryos, 5iLA and NHSM-cultured embryos showed reduced percentages of cells containing H3K27me3 foci in the nuclei (Fig 8C). The results suggest that NHSM indeed delayed *XIST* expression and therefore X-chromosome inactivation.

### 5iLA medium permits embryonic development beyond 9 days

**Fig 9:**
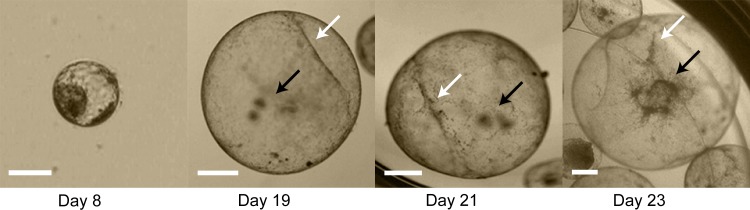
Bovine embryos in 5iLA. Bovine embryos were cultured in 5iLA until day 23. Representative embryos at day 8 until day 23 are shown as indicated. Hypoblast detaching from trophectoderm is indicated (white arrow) as well as disorganized epiblast (black arrow). Scale bar indicates 100μm.

When day 5pf embryos were cultured in 5iLA culture medium it was observed that the embryos remained viable even after day 9 pf while in SOF embryos only remained viable until day 9pf. In vivo in cattle, the blastocyst arrives at the uterus around day 9, but only attaches to the uterine wall around day 24 pf. After zona hatching the trophectoderm elongates and transforms the embryo from a spherical to an ovoid structure, the epiblast together with the hypoblast forms the embryonic disc, and around day 14 mesoderm formation commences [35,36]. 5iLA- cultured embryos increased in size predominantly due to an increase in TE cell number although, in contrast to elongating in vivo embryos, remained spherical even until day 23pf. A distinct embryonic disc was not observed in any of the embryos, instead the epiblast appeared as a disorganized structure (Fig 9). By day 19 pf the epiblast had lost its pluripotent character as evinced by a strong decrease in *NANOG* expression levels (not shown). The hypoblast, identified immunohistochemically by GATA6 expression (not shown) lined the entire trophectoderm and formed a confluent layer that at several parts disconnected from the trophectoderm. Whether any mesoderm differentiation occurred was not clear; Brachyury T expression was not observed using immunohistochemistry (not shown) but this might have been due to species differences between antigen and antibody.

### ICM cell culture in ‘naïve’ media

As the media used were capable of the establishment of naïve human ES cells, ICMs from bovine embryos cultured in these media were isolated and plated in multi-well dishes coated with either CellTak- [37] or a combination of Laminin 521 and E-Cadherin- [25]. In SOF no colony formation could be observed whereas in all ‘naïve’ media and on both coatings all plated ICMs formed colonies and could be passaged after 7 days (Table 3). However, most colonies were not viable after this first passage and only few colonies in the 5iLA medium could be passaged until passage 4. No differences between coatings were observed.

**Table 3:** Bovine ICM culture in 4 media and 2 coatings.

Medium	Coating	ICMs	Colonies after 7 days	Viable at end of passage number (p)				
				p1	p2	p3	p4	
**3iL**	CT	24	24	2		0		
	521/EC	24	24	0				
**5iLA**	CT	24	24	3		3	2	0
	521/EC	24	24	6		2	0	
**NHSM**	CT	24	24	0				
	521/EC	24	24	1		0		
**SOF**	CT	24	0					
	521/EC	24	0					

ICMs were dissected from embryos cultured in 3iL, 5iLA, NHSM or SOF and plated in 24-well plates coated with either Cell-Tak (CT) or Laminin 521 / E-Cadherin (521/EC). After 7 days in their respective condition colonies were passaged.

## Discussion

To establish a pluripotency related transcriptome, bovine embryos were cultured in various naïve human stem cell media from the morula stage onwards. The embryos cultured in NHSM displayed more lipid droplets, particularly in the TE, presumably caused by the serum replacement in NHSM. Preimplantation embryos of ungulates such as cattle contain a relatively high number of lipid droplets, which is most likely used as an energy source needed because of delayed implantation when compared with mouse or human embryos [38]. The ratio of saturated and unsaturated fatty acid species in the lipid droplets may define the quality of the developing embryo [39,40], but at this stage it is not known whether the lipid composition of the droplets changed as a result of NHSM. The increase in numbers of lipid droplets was not reflected by changes in expression of genes associated with lipid metabolism such as Stearoyl CoA desaturases 1 (*SCD1*) or perilipins (*PLIN1*,*2*,*3*,*5*) (Data not shown). Importantly gene expression analysis was performed in isolated ICM while the increase in lipid droplet numbers was primarily observed in the TE, of which gene expression was not analyzed by microarray.

The culture media used in this study had been described for generating naïve human ES cells [9,12,13]. We had anticipated that bovine embryos cultured in these media during the time of ICM/epiblast formation would express higher levels of pluripotency genes than embryos cultured under standard conditions. However, expression levels of core pluripotency genes such as *NANOG*, *SOX2* and *POU5F1* were not enhanced in embryos cultured in these media. At least increased expression of *NANOG* was expected since MAPK inhibition has been shown to increase the percentage of NANOG expressing cells in bovine embryos [1,14,41] and all media investigated contain a MAPK inhibitor. Possibly this effect has been counteracted by FGF in the NHSM whereas for 3iL- and 5iLA-cultured embryos the opposing ingredient remains unknown. Although 5iLA caused a reduced expression of *POU5F1* in the ICM of day 9pf bovine embryos, it is unlikely this resulted in the differentiation into TE as described for mouse [42] since in the TE part of these embryos *NANOG* and *SOX2* expression increased compared to TE from SOF-cultured embryos. Gene expression in day 8pf whole embryos indicative for TE or epiblast differentiation was not altered upon culture in any of the naïve media and PE-specific gene expression was down-regulated. Expression levels of genes marking cell types or developmental stage were inconclusive, probably because pathways involved in pluripotency and differentiation in cattle are regulated differently in mouse. This might be illustrated by low expression levels in bovine ICM of *GBX2* and *NR0B1* in the microarrays performed for this and a previous study [14]. The common marmoset (*Callithrix jacchus*), a primate, also lacks detectable expression of both these pluripotency related genes in the ICM [7]. On the other hand, where the marmoset also lacks *KLF2* and *FBXO15* expression, in the mouse [7] and bovine ICM these genes are expressed. Additionally, *Essrb*/*ESRRB*, *Klf4*/*KLF4* and *Tbx3*/*TBX3* are related to pluripotency in the mouse and primate whereas no differential expression between ICM and TE can be detected in the bovine. These expression profiles indicate subtle differences in the pluripotency network between mouse, primate and cattle.

Genes that were differentially expressed between the ICMs from NHSM- and SOF-cultured embryos could be mapped evenly along the bovine genome except for chromosome 8 and the X-chromosome, both harboring a higher percentage of differentially expressed genes than the other chromosomes. For chromosome 8, most of these genes were located at a small area coding for interferon proteins. Although *IFNT* expression has been detected in the ICM of bovine blastocysts [14,43], its expression is more abundant in the TE [44]. Because of the manual isolation of ICM from TE, it cannot be excluded that ICM samples contained few TE cells. Indeed, we previously determined that about 20% of the cells of the ICM samples had TE characteristics indicated by CDX2 expression [14]. We however think that the presence of a small percentage of TE cells had little effect on the results and the conclusions. For simplicity, we referred to the samples as ICM, rather than ICM +TE. Possibly, TE contamination caused the detected *IFNT* expression in the dissected ICMs. However, the *IFNT* expression was down-regulated more than 6-fold in NHSM, possibly resulting from the JNK-inhibition by SP600125 [45].

On the X-chromosome several differentially expressed genes were distributed over the entire chromosome, representing twice the average amount of differentially expressed genes found on the autosomes. Expression of *XIST* was found to be 1.5-fold down-regulated in NHSM suggesting a reduced or delayed XCI. The possibility that a lower *XIST* expression was a result of a skewed sex-ratio was dismissed by the equal expression of genomic SRY in embryos cultured in SOF and NHSM. Because of the mixture of male and female embryos, the *XIST* expression in female embryos cultured in NHSM is likely even lower.

For rabbit preimplantation embryos it has been described that *XIST* expression is initially expressed in both male and female embryos and in female human embryos both X chromosomes can be active despite *XIST* expression [46]. Exactly how and in which cells X–chromosome inactivation occurs in bovine embryos throughout development is not known. The results with the different culture media indicate however that there is a difference in timing of inactivation for different genes along the X-chromosome.

One of the genes regulated by XCI is *HPRT1* [46] and its expression was 2-fold up-regulated in NHSM. Other genes on the X-chromosome and known to be regulated by XCI such as *G6PD* and *SMCX* were not significantly differentially expressed. This might be a result of incomplete X-chromosome coverage or absence of XCI despite the expression of *XIST*, like described for human blastocysts [46]. Genes known to escape XCI in bovine including *ZFX*, *UTX*, *MED14* (*CRSP2*) and *UBA1* were either not differentially expressed or slightly down-regulated in NHSM. Only little is known about XCI in bovine embryos [47]. However, in elongated in vivo day 14pf female embryos, XCI is present [47,48]. Quantitative RT-PCR data indicated a further reduction in *XIST* expression in day 9pf ICMs compared to day 8pf embryos, both cultured in NHSM. Similar to the day 9pf ICMs, *HPRT1* was more than 2-fold up-regulated in day 8pf blastocysts although its lack of significance might be caused by the presence of all TE cells compared to the isolated ICMs. In 3iL and 5iLA *XIST* expression was even further reduced as in NHSM, although without differential expression of *HPRT1* compared to SOF-cultured day 8pf bovine embryos. Besides *XIST* coverage of the inactivated X-chromosome abundant H3K27 trimethylation on one of the X-chromosomes plays a key role in XCI [34,49,50]. In female embryos cultured in SOF, NHSM and 5iLA, cells could be identified with a distinct H3K27me3 spot, indicating X chromosome inactivation. In parallel with embryos from other eutherian mammals it is expected that in early embryonic and trophoblast cells the paternal X-chromosome is inactivated, while in pluripotent cells of the ICM epiblast both X-chromosomes are active [33]. Indeed this phenomena has been described in bovine placental tissue [51]. In SOF-cultured female embryos around 40% of the cells exhibited H3K27me3 foci which is slightly higher than the 25% and 21% described in human [52] and bovine [53] trophoblast cells respectively. However, these data suggest that not all TE cells experience Xi at this stage.

Embryos cultured in NHSM and 5iLA showed lower percentages of cells with H3K27me3 foci. Since the H3K27me3 pattern does not allow distinguishing between imprinted and random Xi and we were not able to distinguish ICM from TE in the stained embryos, it cannot be excluded that this reduction was also due to reduced Xi in TE cells. Independently, the reduced percentages of cells with H3K27me3 foci indicate a delayed or a reduced establishment of XCI in bovine embryos cultured in ‘naïve’ conditions.

Taken together, the results indicate that the conditions capable of capturing pluripotency from human ICM cells are unfit for enhancing pluripotency in the ICM of the bovine blastocyst. Signaling pathways driving lineage commitment and segregation in the ICM of cattle and primate embryos is poorly defined. Therefore, it is still possible that for these species other pluripotency pathways are in play and the genes, signaling pathways and factors are so far unknown. The microarray and qRT-PCR data obtained might aid as a starting point to identify these signaling pathways. In addition, the prolonged in vitro culture of bovine embryos cultured in 5iLA can be useful for studying events such as extraembryonic mesoderm formation and gastrulation. Whether these processes indeed occur in the in vitro cultured embryos requires further investigation.

## Supporting information

S1 Fig Cell number and ICM composition per embryo. Embryos cultured in SOF, SOF+KOSR, 3iL, 5iLA or NHSM were stained with DAPI and cell number per embryo determined after 8 days (A) and 9 days (B) post fertilization. Averaged results are in Fig 1E and 1F. ICM part per embryo was determined by density and size of cells and an ellipse was positioned to define a region to count and calculate percentage of GATA6 (red) positive cells, NANOG (green) positive cells, double positive (orange) or double negative (blue) cells in the same single embryo cultured until day 8pf (C) or day 9pf (D).
(TIF)

S2 Fig Expression levels in day 8pf embryos. Relative expression of genes marking TE, PE and epiblast, involved in pluripotency and implicated in methylation or differentiation as determined by qRT-PCR is plotted with respect to expression in SOF-cultured embryos (set at 1; dashed line) for 3iL (blue), 5iLA (red) and NHSM (green). Significant differences (p<0.05) are indicated by different letters.
(TIF)

S1 File H3K27me3 embryo. Stack view of representative H3K27me3 stained embryo per slice (same embryo as Fig 8A).
(TIF)

S2 File H3K27me3 staining. Video generated in IMARIS of an H3K27me3 stained representative embryo (same embryo as in Fig 8A).
(MP4)

S1 Table Details of primers used for (qRT-)PCR. Primer sequences and annealing temperatures (Ta) for gene expression determination. Of primer sets not published before Genbank accession numbers are given. Otherwise references of publication are given. ^G^ Indicates primer sets used for genomic detection. * Indicates primer sets used for normalization.
(DOCX)

S2 Table Differentially expressed genes after microarray analysis. All genes differentially expressed (1.5-fold; p<0.05) according to microarray data are listed with their chromosome. Negative fold change (FC) values indicate lower expression levels in NHSM ICMs and positive values are at higher expression levels in NHSM ICMs compared with SOF ICMs.
(DOCX)
