# Supplementary figures and images for: Characterization of bovine embryos cultured under conditions appropriate for sustaining human naïve pluripotency
Source: PLoS One. 2017 Feb 27;12(2):e0172920. doi: 10.1371/journal.pone.0172920 (PMC5328396; doi:10.1371/journal.pone.0172920)

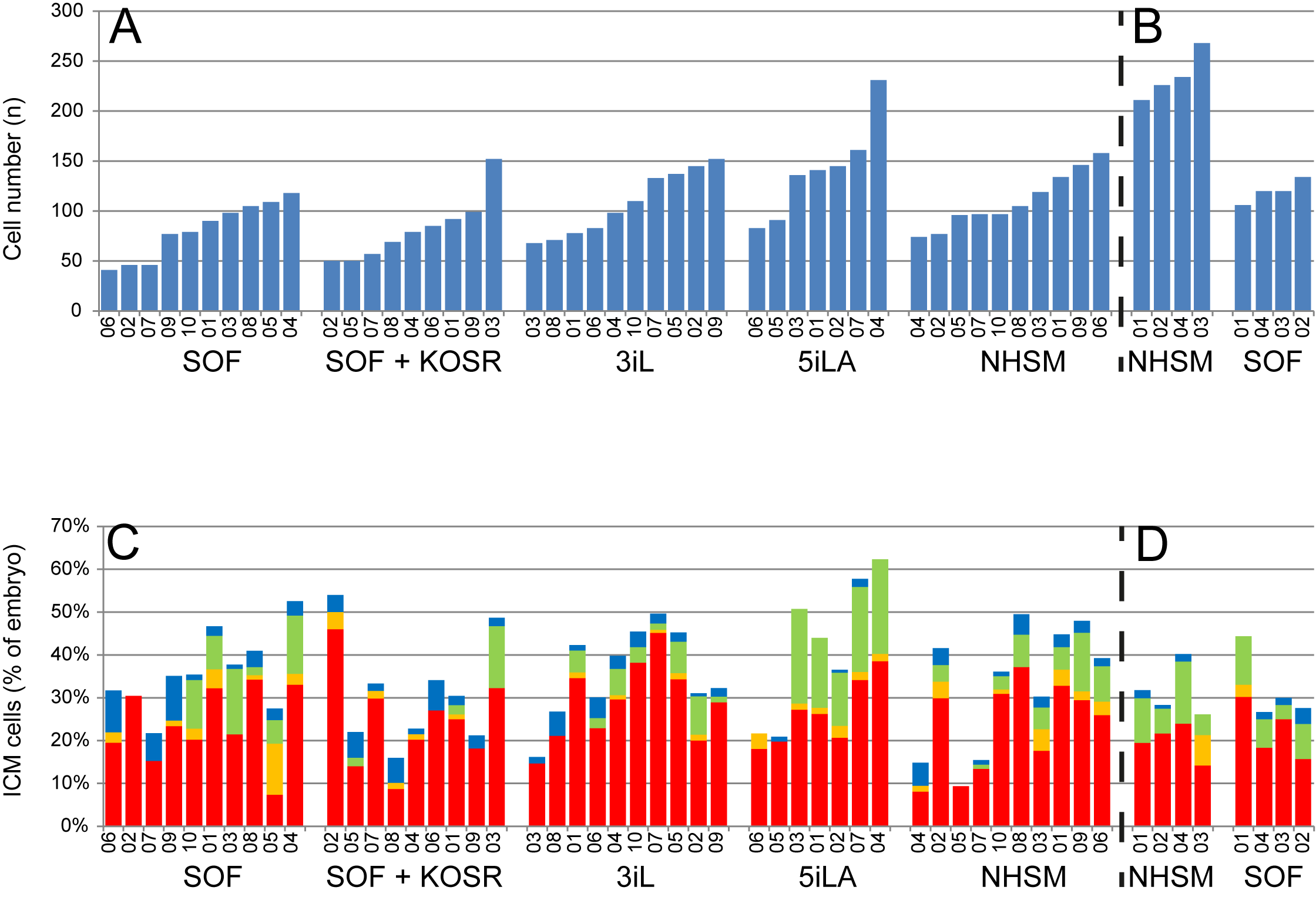

Supplement: S1 Fig — Embryos cultured in SOF, SOF+KOSR, 3iL, 5iLA or NHSM were stained with DAPI and cell number per embryo determined after 8 days (A) and 9 days (B) post fertilization. Averaged results are in Fig 1E and 1F. ICM part per embryo was determined by density and size of cells and an ellipse was positioned to define a region to count and calculate percentage of GATA6 (red) positive cells, NANOG (green) positive cells, double positive (orange) or double negative (blue) cells in the same single embryo cultured until day 8pf (C) or day 9pf (D). (TIF) [file pone.0172920.s001.tif]

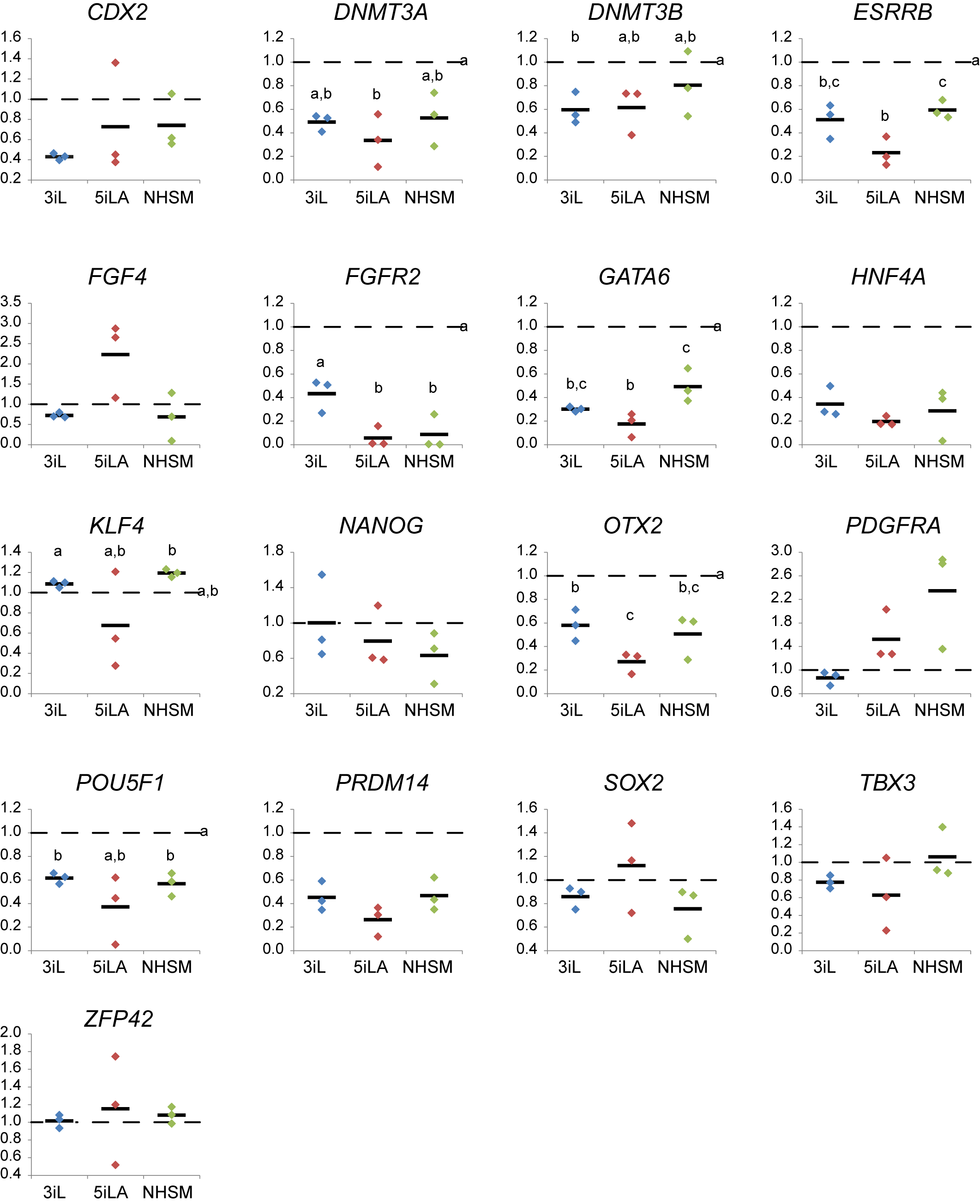

Supplement: S2 Fig — Relative expression of genes marking TE, PE and epiblast, involved in pluripotency and implicated in methylation or differentiation as determined by qRT-PCR is plotted with respect to expression in SOF-cultured embryos (set at 1; dashed line) for 3iL (blue), 5iLA (red) and NHSM (green). Significant differences (p<0.05) are indicated by different letters. (TIF) [file pone.0172920.s002.tif]
