# Supplementary material for: Characterization of bovine embryos cultured under conditions appropriate for sustaining human naïve pluripotency
Source: PLoS One. 2017 Feb 27;12(2):e0172920. doi: 10.1371/journal.pone.0172920 (PMC5328396; doi:10.1371/journal.pone.0172920)
Supplement: S1 Table — Primer sequences and annealing temperatures (Ta) for gene expression determination. Of primer sets not published before Genbank accession numbers are given. Otherwise references of publication are given. G Indicates primer sets used for genomic detection. * Indicates primer sets used for normalization. (DOCX) [file pone.0172920.s005.docx]

| **Gene** | **Description** | Acc.# (Genbank) or reference | **Primer forward sequence** | **Primer reverse sequence** | **Ta (°C)** |
| --- | --- | --- | --- | --- | --- |
| CDC42 ^G^* | Cell division cycle 42 | NM_001046332 | GTGCCTGAGATAACTCACCA | GGAGTGATAGGCTTCTGCTT | 61 |
| CDX2 | Caudal type homeobox 2 | [14] | AACCTGTGCGAGTGGA | GCGACTGTAGTGAAACTCC | 60 |
| DDX3Y | DEAD-box helicase 3, Y-linked | [54] | GGACGTGTAGGAAACCTTGG | GCCAGAACTGCTACTTTGTCG | 60 |
| DNMT3a | DNA (cytosine-5-)-methyltransferase 3 alpha | [41] | CTGGTGCTGAAGGACTTGGGC | CAGAAGAAGGGGCGGTCATC | 57 |
| DNMT3b | DNA (cytosine-5-)-methyltransferase 3 beta | [41] | CCGCAGATCAAGCTCAC | GTTATTTCGGGTTCGGAC | 60 |
| ESRRB | Estrogen-related receptor beta | XM_606860 | ATGAAATGCCTCAAAGTGG | TCAGAGCCTTGATGTCG | 61 |
| FGF4 | Fibroblast growth factor 4 | [41] | TACGGCTCGCCTTTCTTCAC | TTCTTGGCCTTGCCGTTCTT | 60 |
| FGFR2 | Fibroblast growth factor receptor 2 | [41] | TGTAAGAGAAAAGGAGATCCCA | GTTGAAGAGAGGCGAGTTG | 56 |
| GAPDH | Glyceraldehyde 3-phosphate dehydrogenase | AJ000039 | AGGCATCACCATCTTCCAG | GGCGTGGACAGTGGTCATAA | 61 |
| GATA6 | GATA binding protein 6 | XM_002697727 | GTGAACTGCGGCTCCATCCA | AGGCATTGCACACAGGCTCG | 67 |
| GCA | Grancalcin, EF-hand calcium binding protein | XM_002685376 | GTCCTCAAACAGTAACTACC | ACTCACAGTATTTCTTCAGC | 62 |
| GREM1 ^G^* | Gremlin 1, DAN family BMP antagonist | NM_001082450 | CATCAACCGCTTCTGCTACG | TGGCTGGAGTTCAGGACAGT | 64 |
| HNF4a | Hepatocyte nuclear factor 4, alpha | [55] | CGGGCCACGGGCAAACACTA | CACCACGCACTGCCGGCTAA | 58 |
| HPRT1 | Hypoxanthine phosphoribosyltransferase 1 | [56] | TGCTGAGGATTTGGAGAAGG | CAACAGGTCGGCAAAGAACT | 58 |
| ID1 | Inhibitor of DNA binding 1, dominant negative helix-loop-helix protein | [55] | GTGTTCCAGCGGACGATCGT | GTGGCCGCCAATCGTTCTTG | 58 |
| IFNT | Interferon-tau 3g | [14] | GATGACTCTCGCTGACTAAGATGC | CTGCTGACAAAGTATCGGCTAAAG | 60 |
| KLF4 | Kruppel-like factor 4 | NM_001105385 | TCTCATCTCAAGGCACACCTGCG | GCGGGCAAACTTCCACCCAC | 61 |
| NANOG | Nanog homeobox | [14] | GAGAGCACAGAGAAGGAAGA | CTGGTGGTAGGAATAGAAGC | 60 |
| OTX2 | Orthodenticle homeobox 2 | XM_005211840 | GGGCTGAGTCTGACCACTTC | AACCATACCTGTACCCTGGACT | 58 |
| PDGFRa | Platelet-derived growth factor receptor, alpha polypeptide | [41] | GCCAACCAGATGTGAGGTGA | AAAGACCACGCTGGCAGTAA | 60 |
| POU5F1 | POU class 5 homeobox 1 | NM_174580 | TAGCCACATCGCCCAGCAGC | GAAAGGAGACCCAGCAGCCTCA | 62 |
| PPIA | peptidylprolyl isomerase A (cyclophilin A) | NM_178320 | CTGCTTTCACAGAATAATTCCG | TTGCCATCCAACCACTC | 57 |
| PRDM14 | PR domain containing 14 | [41] | ATTTTCGTTCCGCCGCCCCC | TCAGCCCCGGCGCTATCGGT | 60 |
| RPL15* | Ribosomal protein L15 mRNA | AY786141 | CACAAGTTCCACCACACTATTGG | TGGAGAGTATTGCGCCTTCTC | 61 |
| SDHA* | Succinate dehydrogenase complex, subunit A, flavoprotein (Fp) | [56] | GCAGAACCTGATGCTTTGTG | CGTAGGAGAGCGTGTGCTT | 64 |
| SOX2 | SRY (sex determining region Y)-box 2 | NM_001105463 | CCATGCAGGTTGACATCGT | ACACAACTACGGAAACTAAAAGTGG | 60 |
| SRY ^G^ | Sex determining region Y | NM_001014385 | ACAGTCATAGCGCAAATGATCAGTG | GGGTTGCATAGTATTGAAGAGTCTGC | 67 |
| TBX3 | T-box 3 | [41] | CGGATTTACTTTGGCCTTCCC | CTGGTATGCAGTCACAGCGA | 60 |
| UBB * | Bos taurus polyubiquitin | NM_174133 | CTGACCGGCAAGACCATCAC | AGATGAGCCTCTGCTGGTCG | 61 |
| VIM | Vimentin | [57] | ATGAAGGAAGAGATGGCTCGTCAC | TCCAGATTGGTTTCCCTCAGGTTC | 60 |
| XIST | X (inactive)-specific transcript | NR_001464 | AGCATTGCTTAGCATGGCTC | TGGCTGTGACCGATTCTACC | 60 |
| YWHAZ* | Tyrosine-3-monooxygenase/tryptophan 5-monooxygenase activation protein zeta polypeptide | [56] | GCATCCCACAGACTATTTCC | GCAAAGACAATGACAGACCA | 63 |
| ZFP42 | ZFP42 zinc finger protein | [58] | TGCCTGTCCTCACAACGGATGC | AGTGTGGGTGCGCACGTGTG | 60 |

Supporting references for supplemental Table 1:

54. Hamilton CK, Combe A, Caudle J, Ashkar FA, Macaulay AD, Blondin P, et al. A novel approach to sexing bovine blastocysts using male-specific gene expression. Theriogenology. 2012;77: 1587-1596. doi: 10.1016/j.theriogenology.2011.11.027.

55. Nagatomo H, Kagawa S, Kishi Y, Takuma T, Sada A, Yamanaka K, et al. Transcriptional Wiring for Establishing Cell Lineage Specification at the Blastocyst Stage in Cattle1. Biol Reprod. 2013;88: 158. doi: 10.1095/biolreprod.113.108993.

56. Goossens K, Van Poucke M, Van Soom A, Vandesompele J, Van Zeveren A, Peelman LJ. Selection of reference genes for quantitative real-time PCR in bovine preimplantation embryos. BMC Dev Biol. 2005;5: 27. doi: 10.1186/1471-213X-5-27.

57. Liggett TE, Griffiths TD, Gaillard ER. Isolation and characterization of a spontaneously immortalized bovine retinal pigmented epithelial cell line. BMC Cell Biol. 2009;10: 33. doi: 10.1186/1471-2121-10-33.

58. Huang B, Li T, Alonso-Gonzalez L, Gorre R, Keatley S, Green A, et al. A virus-free poly-promoter vector induces pluripotency in quiescent bovine cells under chemically defined conditions of dual kinase inhibition. PLoS ONE. 2011;6: e24501. doi: 10.1371/journal.pone.0024501.
