# Supplementary material for: Characterization of bovine embryos cultured under conditions appropriate for sustaining human naïve pluripotency
Source: PLoS One. 2017 Feb 27;12(2):e0172920. doi: 10.1371/journal.pone.0172920 (PMC5328396; doi:10.1371/journal.pone.0172920)
Supplement: S2 Table — All genes differentially expressed (1.5-fold; p<0.05) according to microarray data are listed with their chromosome. Negative fold change (FC) values indicate lower expression levels in NHSM ICMs and positive values are at higher expression levels in NHSM ICMs compared with SOF ICMs. (DOCX) [file pone.0172920.s006.docx]

**Supplemental Table 2 Differentially expressed genes.**

| **Gene Symbol (NCBI)** | **Description** | **Chr** | **NHSM vs SOF (FC)** |
| --- | --- | --- | --- |
| IFN-tau-c1 | interferon-tau 3g precursor [Source:RefSeq peptide;Acc:NP_001161751] | 8 | -6.75 |
| CT47B1 | cancer/testis antigen family 147, member B1 [Source:RefSeq peptide;Acc:NP_001092672] | X | 5.45 |
| DUSP4 | Bos taurus dual specificity phosphatase 4 (DUSP4), mRNA. [Source:RefSeq mRNA;Acc:NM_001206953] | 27 | -5.15 |
| ENSBTAG00000034326 | Uncharacterized protein [Source:UniProtKB/TrEMBL;Acc:E1BPH2] | 8 | -4.71 |
| ENSBTAG00000046119 | Uncharacterized protein [Source:UniProtKB/TrEMBL;Acc:G3N350] | 8 | -4.67 |
| LOC100847720 | Uncharacterized protein [Source:UniProtKB/TrEMBL;Acc:G3N2M0] | 8 | -4.56 |
| ENSBTAG00000034320 | Novel pseudogene | 8 | -4.03 |
| LOC101905249 | melanoma-associated antigen B1-like | X | 3.97 |
| ABCC2 | Uncharacterized protein [Source:UniProtKB/TrEMBL;Acc:F1MIP7] | 26 | -3.76 |
| ENSBTAG00000039827 | Uncharacterized protein [Source:UniProtKB/TrEMBL;Acc:F1MT30] | 8 | -3.69 |
| DAZL | deleted in azoospermia-like [Source:RefSeq peptide;Acc:NP_001075194] | 1 | 3.36 |
| ENSBTAG00000046815 | Uncharacterized protein [Source:UniProtKB/TrEMBL;Acc:E1BMT4] | 8 | -3.35 |
| LOC618806 | melanoma-associated antigen 10-like [Source:RefSeq peptide;Acc:NP_001193285] | 10 | 3.34 |
| GPC4 | glypican-4 precursor [Source:RefSeq peptide;Acc:NP_001192713] | X | -3.21 |
| ENSBTAG00000045727 | Novel pseudogene | 14 | -3.19 |
| ACTA1 | Actin, alpha skeletal muscle [Source:UniProtKB/Swiss-Prot;Acc:P68138] | 28 | 3.17 |
| PDYN | proenkephalin-B preproprotein [Source:RefSeq peptide;Acc:NP_776564] | 13 | -3.13 |
| LOC100336885 | Uncharacterized protein [Source:UniProtKB/TrEMBL;Acc:G3N3M8] | 8 | -3.03 |
| TKTL1 | Transketolase-like protein 1 [Source:UniProtKB/Swiss-Prot;Acc:Q2NL26] | X | -3.00 |
| LOC520085 | Bos taurus similar to melanoma antigen (LOC520085), mRNA /cds=p(1,870) /gb=XM_598316 /gi=194668428 /ug=Bt.104735 /len=870 | X | 2.99 |
| QPRT | nicotinate-nucleotide pyrophosphorylase [Source:RefSeq peptide;Acc:NP_001030523] | 25 | -2.99 |
| PLAU | Urokinase-type plasminogen activator Urokinase-type plasminogen activator long chain A Urokinase-type plasminogen activator short chain A Urokinase-type plasminogen activator chain B [Source:UniProtKB/Swiss-Prot;Acc:Q05589] | 28 | -2.94 |
| SPRY4 | sprouty homolog 4 (Drosophila) | 7 | -2.93 |
| SLC13A4 | solute carrier family 13 member 4 [Source:RefSeq peptide;Acc:NP_001070352] | 4 | -2.87 |
| STC1 | Stanniocalcin-1 [Source:UniProtKB/Swiss-Prot;Acc:Q9N0T1] | 8 | 2.85 |
| XDH | xanthine dehydrogenase/oxidase [Source:RefSeq peptide;Acc:NP_776397] | 11 | 2.85 |
| FSCN1 | fascin [Source:RefSeq peptide;Acc:NP_001030217] | 25 | 2.78 |
| HAS2 | hyaluronan synthase 2 [Source:RefSeq peptide;Acc:NP_776504] | 14 | -2.77 |
| TFRC | Transferrin receptor protein 1 [Source:RefSeq peptide;Acc:NP_001193506] | 1 | 2.73 |
| MAFF | Transcription factor MafF [Source:UniProtKB/Swiss-Prot;Acc:A7YY73] | 5 | -2.73 |
| LOC618801 | 3-hydroxy-3-methylglutaryl-coenzyme A reductase [Source:UniProtKB/Swiss-Prot;Acc:A7Z064] | 10 | -2.69 |
| LOC786942 | uncharacterized LOC786942 | X | 2.66 |
| LOC618801 | Uncharacterized protein [Source:UniProtKB/TrEMBL;Acc:G3N3U4] | 8 | -2.65 |
| LDLR | low-density lipoprotein receptor precursor [Source:RefSeq peptide;Acc:NP_001160002] | 7 | -2.65 |
| RGS20 | Regulator of G-protein signaling 20 [Source:UniProtKB/Swiss-Prot;Acc:P79348] | 14 | -2.65 |
| BC108185.1 | Bos taurus cDNA clone IMAGE:8059386. | X | 2.62 |
| SLC7A3 | cationic amino acid transporter 3 [Source:RefSeq peptide;Acc:NP_001071487] | X | -2.62 |
| ARRDC4 | arrestin domain-containing protein 4 [Source:RefSeq peptide;Acc:NP_001178963] | 21 | 2.61 |
| LOC100847577 | P antigen family member 3-like | X | 2.56 |
| ENSBTAG00000034294 | Novel pseudogene | 8 | -2.55 |
| PLET1 | Placenta-expressed transcript 1 protein [Source:UniProtKB/Swiss-Prot;Acc:A5D7U1] | 15 | 2.54 |
| C9H6orf165 | UPF0704 protein C6orf165 homolog [Source:UniProtKB/Swiss-Prot;Acc:Q29RL1] | 9 | 2.53 |
| LDHA | L-lactate dehydrogenase A chain [Source:UniProtKB/Swiss-Prot;Acc:P19858] | 29 | 2.53 |
| MS4A8B | membrane-spanning 4-domains, subfamily A, member 8B [Source:RefSeq peptide;Acc:NP_001029228] | 29 | -2.49 |
| LOC101904389 | uncharacterized LOC101904389 | 6 | 2.49 |
| GARNL3 | GTPase-activating Rap/Ran-GAP domain-like protein 3 [Source:RefSeq peptide;Acc:NP_001071340] | 11 | 2.48 |
| ACSL4 | Acyl-CoA synthetase long-chain family member 4 [Source:HGNC Symbol;Acc:HGNC:3571] | X | -2.48 |
| ARSA | Arylsulfatase A [Source:UniProtKB/Swiss-Prot;Acc:Q08DD1] | 5 | -2.46 |
| C26H10orf96 | UPF0628 protein C10orf96 homolog [Source:RefSeq peptide;Acc:NP_001069301] | 26 | 2.46 |
| BC149677.1 | Bos taurus cDNA clone IMAGE:8057572. | X | 2.43 |
| CYP1A1 | Uncharacterized protein [Source:UniProtKB/TrEMBL;Acc:F1MM10] | 21 | -2.43 |
| NGFRAP1 | Protein BEX3 [Source:UniProtKB/Swiss-Prot;Acc:Q3ZBJ6] | X | 2.43 |
| MAGEB16 | melanoma antigen family B, 16 [Source:RefSeq peptide;Acc:NP_001098869] | X | 2.43 |
| L1CAM | neural cell adhesion molecule L1 precursor [Source:RefSeq peptide;Acc:NP_001179364] | X | -2.42 |
| HMGCS1 | 3-hydroxy-3-methylglutaryl-CoA synthase 1 (soluble) [Source:HGNC Symbol;Acc:HGNC:5007] | 20 | -2.40 |
| DNASE1L3 | deoxyribonuclease gamma precursor [Source:RefSeq peptide;Acc:NP_001192653] | 22 | -2.40 |
| DUSP6 | Dual specificity protein phosphatase 6 [Source:UniProtKB/Swiss-Prot;Acc:Q2KJ36] | 5 | -2.40 |
| MCC | colorectal mutant cancer protein [Source:RefSeq peptide;Acc:NP_001076992] | 10 | 2.39 |
| LOC786718 | uncharacterized LOC786718 | X | 2.36 |
| SLC20A2 | sodium-dependent phosphate transporter 2 [Source:RefSeq peptide;Acc:NP_001073749] | 27 | -2.35 |
| CLIC6 | Uncharacterized protein [Source:UniProtKB/TrEMBL;Acc:E1BAI4] | 1 | -2.35 |
| LRRC8E | leucine-rich repeat-containing protein 8E [Source:RefSeq peptide;Acc:NP_001192551] | 7 | -2.34 |
| SERPINA5 | Plasma serine protease inhibitor [Source:UniProtKB/Swiss-Prot;Acc:Q9N2I2] | 21 | -2.34 |
| DHRS9 | Dehydrogenase/reductase SDR family member 9 [Source:UniProtKB/Swiss-Prot;Acc:Q8HYR6] | 2 | 2.32 |
| LOC618023 | cancer/testis antigen 1-like | X | 2.31 |
| PAGE4 | G antigen family C member 1 [Source:RefSeq peptide;Acc:NP_001075049] | X | 2.30 |
| NUCB2 | nucleobindin-2 precursor [Source:RefSeq peptide;Acc:NP_001068849] | 15 | -2.29 |
| TUBB2B | tubulin beta-2B chain [Source:RefSeq peptide;Acc:NP_001003900] | 23 | 2.29 |
| BCKDHA | 2-oxoisovalerate dehydrogenase subunit alpha, mitochondrial precursor [Source:RefSeq peptide;Acc:NP_776931] | 18 | -2.28 |
| PIM1 | Bos taurus pim-1 oncogene (PIM1), mRNA. [Source:RefSeq mRNA;Acc:NM_174144] | 23 | -2.27 |
| SULT1E1 | estrogen sulfotransferase [Source:RefSeq peptide;Acc:NP_803454] | 6 | 2.27 |
| PTER | Phosphotriesterase-related protein [Source:UniProtKB/Swiss-Prot;Acc:A6QLJ8] | 13 | 2.27 |
| ENSBTAG00000012835 | Novel pseudogene | X | 2.26 |
| LOC783543 | Eso3 protein-like | X | 2.26 |
| SLC6A1 | sodium- and chloride-dependent GABA transporter 1 [Source:RefSeq peptide;Acc:NP_001071304] | 22 | -2.24 |
| TUBA4A | Tubulin alpha-4A chain [Source:UniProtKB/Swiss-Prot;Acc:P81948] | 2 | 2.23 |
| RBM38 | RNA-binding protein 38 [Source:RefSeq peptide;Acc:NP_001192952] | 13 | 2.23 |
| EGR1 | early growth response protein 1 [Source:RefSeq peptide;Acc:NP_001039340] | 7 | -2.21 |
| RPTOR | regulatory-associated protein of mTOR [Source:RefSeq peptide;Acc:NP_001179059] | 19 | -2.21 |
| LY6K | Uncharacterized protein [Source:UniProtKB/TrEMBL;Acc:F1N4E6] | 14 | 2.21 |
| THBS4 | Thrombospondin-4 [Source:UniProtKB/Swiss-Prot;Acc:Q3SWW8] | 10 | -2.21 |
| COL5A2 | Uncharacterized protein [Source:UniProtKB/TrEMBL;Acc:F1N2Y2] | 2 | -2.21 |
| LRP5 | Uncharacterized protein [Source:UniProtKB/TrEMBL;Acc:F1N1K8] | 29 | -2.20 |
| CDH2 | Cadherin-2 [Source:UniProtKB/Swiss-Prot;Acc:P19534] | 24 | -2.19 |
| PRPH | Peripherin [Source:UniProtKB/Swiss-Prot;Acc:A6QQJ3] | 5 | -2.19 |
| KLF15 | Krueppel-like factor 15 [Source:RefSeq peptide;Acc:NP_001075894] | 22 | -2.18 |
| IFI30 | gamma-interferon-inducible lysosomal thiol reductase precursor [Source:RefSeq peptide;Acc:NP_001094721] | 7 | -2.18 |
| HPRT1 | Hypoxanthine-guanine phosphoribosyltransferase [Source:UniProtKB/Swiss-Prot;Acc:Q3SZ18] | X | 2.17 |
| CYP1B1 | cytochrome P450, family 1, subfamily B, polypeptide 1 (CYP1B1), mRNA. [Source:RefSeq mRNA;Acc:NM_001192294] | 11 | -2.14 |
| FRAT2 | Uncharacterized protein [Source:UniProtKB/TrEMBL;Acc:G3N174] | 26 | -2.13 |
| DIP2C | disco-interacting protein 2 homolog C [Source:RefSeq peptide;Acc:NP_001193002] | 13 | 2.13 |
| SLC27A6 | long-chain fatty acid transport protein 6 [Source:RefSeq peptide;Acc:NP_001094639] | 7 | 2.12 |
| EID2 | EP300-interacting inhibitor of differentiation 2 [Source:UniProtKB/Swiss-Prot;Acc:Q17QW4] | 18 | 2.12 |
| LOC781498 | adaptor-related protein complex 1, sigma 2 subunit pseudogene | 4 | -2.12 |
| STMN2 | stathmin-2 [Source:RefSeq peptide;Acc:NP_001029794] | 14 | -2.11 |
| WC1 | Uncharacterized protein [Source:UniProtKB/TrEMBL;Acc:F1N6N9] | 5 | 2.10 |
| TEX12 | testis-expressed sequence 12 protein [Source:RefSeq peptide;Acc:NP_001029435] | 15 | 2.10 |
| ATP12A | Uncharacterized protein [Source:UniProtKB/TrEMBL;Acc:F1N1K4] | 12 | -2.08 |
| bta-mir-2900 | bta-mir-2900 Source: miRBase MI0013075 | 18 | 2.07 |
| BT.50520 | Uncharacterized protein [Source:UniProtKB/TrEMBL;Acc:G3N2A3] | X | 2.07 |
| ENSBTAG00000039847 | Uncharacterized protein [Source:UniProtKB/TrEMBL;Acc:E3W9A0] | 5 | 2.05 |
| BEX5 | Protein BEX5 [Source:UniProtKB/Swiss-Prot;Acc:Q3ZBJ9] | X | 2.05 |
| PSPH | Phosphoserine phosphatase [Source:UniProtKB/Swiss-Prot;Acc:Q2KHU0] | 25 | 2.04 |
| MGC133764 | melanoma antigen family B-like [Source:RefSeq peptide;Acc:NP_001073841] | X | 2.03 |
| SLC35D2 | UDP-N-acetylglucosamine/UDP-glucose/GDP-mannose transporter [Source:RefSeq peptide;Acc:NP_001193195] | 8 | -2.03 |
| AKAP2 | A-kinase anchor protein 2 [Source:RefSeq peptide;Acc:NP_001179963] | 8 | -2.02 |
| LOC100126053 | A6QQ62_BOVIN LOC100126053 protein | X | 2.02 |
| CD163L1 | antigen WC1.1 precursor [Source:RefSeq peptide;Acc:NP_788824] | 5 | 2.02 |
| ENSBTAG00000039540 | uncharacterized protein LOC616319 [Source:RefSeq peptide;Acc:NP_001094724] | 3 | 2.02 |
| RIMKLB | ribosomal modification protein rimK-like family member B | 5 | -2.01 |
| VIM | Vimentin [Source:UniProtKB/Swiss-Prot;Acc:P48616] | 13 | -2.01 |
| ST6GAL1 | ST6 beta-galactosamide alpha-2,6-sialyltranferase 1 (ST6GAL1), mRNA. [Source:RefSeq mRNA;Acc:NM_177517] | 1 | 2.01 |
| XRCC2 | DNA repair protein XRCC2 [Source:RefSeq peptide;Acc:NP_001095824] | 4 | 2.01 |
| ZIC2 | zinc finger protein ZIC 2 [Source:RefSeq peptide;Acc:NP_001193295] | 12 | -2.01 |
| SIPA1L1 | Uncharacterized protein [Source:UniProtKB/TrEMBL;Acc:F1MXV4] | 10 | -2.01 |
| UCHL1 | Ubiquitin carboxyl-terminal hydrolase isozyme L1 [Source:UniProtKB/Swiss-Prot;Acc:P23356] | 6 | 2.00 |
| WSB1 | Uncharacterized protein [Source:UniProtKB/TrEMBL;Acc:E1BBZ9] | 19 | -2.00 |
| HSPB8 | Heat shock protein beta-8 [Source:UniProtKB/Swiss-Prot;Acc:Q5EAC9] | 17 | 1.99 |
| C12ORF63 | Uncharacterized protein [Source:UniProtKB/TrEMBL;Acc:E1BPJ0] | 5 | -1.99 |
| ALDH3A2 | fatty aldehyde dehydrogenase [Source:RefSeq peptide;Acc:NP_001095454] | 19 | -1.99 |
| APBA2 | amyloid beta A4 precursor protein-binding family A member 2 [Source:RefSeq peptide;Acc:NP_001071389] | 21 | -1.99 |
| UPP1 | uridine phosphorylase 1 [Source:RefSeq peptide;Acc:NP_001092446] | 4 | -1.98 |
| MYBPC3 | myosin-binding protein C, cardiac-type [Source:RefSeq peptide;Acc:NP_001070004] | 15 | -1.98 |
| EVI2B | protein EVI2B [Source:RefSeq peptide;Acc:NP_001092636] | 19 | -1.97 |
| KIAA0922 | Transmembrane protein 131-like [Source:UniProtKB/Swiss-Prot;Acc:Q08DV9] | 17 | -1.97 |
| PRM1 | Sperm protamine P1 [Source:UniProtKB/Swiss-Prot;Acc:P02318] | 25 | 1.97 |
| HBA | Hemoglobin subunit alpha [Source:UniProtKB/Swiss-Prot;Acc:P01966] | 25 | 1.97 |
| LRAT | lecithin retinol acyltransferase [Source:RefSeq peptide;Acc:NP_803469] | 17 | -1.96 |
| GPR1 | G-protein coupled receptor 1 [Source:RefSeq peptide;Acc:NP_001193474] | 2 | -1.96 |
| SC4MOL | methylsterol monooxygenase 1 [Source:RefSeq peptide;Acc:NP_001092333] | 17 | -1.95 |
| RRAGD | Bos taurus Ras-related GTP binding D (RRAGD), mRNA. [Source:RefSeq mRNA;Acc:NM_001192828] | 9 | -1.95 |
| MAPK13 | mitogen-activated protein kinase 13 [Source:RefSeq peptide;Acc:NP_001014947] | 23 | 1.95 |
| RTP3 | Uncharacterized protein [Source:UniProtKB/TrEMBL;Acc:E1BAA1] | 22 | 1.95 |
| TDGF1 | teratocarcinoma-derived growth factor 1 precursor [Source:RefSeq peptide;Acc:NP_001073827] | 22 | -1.95 |
| RNF145 | RING finger protein 145 [Source:RefSeq peptide;Acc:NP_001095638] | 7 | -1.95 |
| NXF3 | nuclear RNA export factor 3 [Source:RefSeq peptide;Acc:NP_001071520] | X | 1.94 |
| MYL7 | Uncharacterized protein [Source:UniProtKB/TrEMBL;Acc:F1N2V9] | 4 | 1.94 |
| NRBP2 | nuclear receptor-binding protein 2 [Source:RefSeq peptide;Acc:NP_001071316] | 14 | -1.93 |
| DIRAS3 | GTP-binding protein Di-Ras3 [Source:RefSeq peptide;Acc:NP_001029387] | 3 | 1.92 |
| LOC101902201 | cancer/testis antigen 1-like | X | 1.92 |
| GPR64 | Uncharacterized protein [Source:UniProtKB/TrEMBL;Acc:F1MRZ3] | X | -1.92 |
| RASD1 | dexamethasone-induced Ras-related protein 1 [Source:RefSeq peptide;Acc:NP_001193190] | 19 | 1.92 |
| PAG11 | pregnancy-associated glycoprotein 11 [Source:RefSeq peptide;Acc:NP_788796] | 29 | 1.92 |
| Unknown 1 | unknown | 23 | 1.92 |
| GCA | Uncharacterized protein [Source:UniProtKB/TrEMBL;Acc:F1N7I6] | 2 | 1.92 |
| ASGR1 | asialoglycoprotein receptor 1 [Source:RefSeq peptide;Acc:NP_001032679] | 19 | 1.91 |
| NXT2 | NTF2-related export protein 2 [Source:UniProtKB/Swiss-Prot;Acc:A6QNX3] | X | 1.90 |
| S100B | Protein S100-B [Source:UniProtKB/Swiss-Prot;Acc:P02638] | 1 | 1.90 |
| RBM20 | RNA binding motif protein 20 [Source:HGNC Symbol;Acc:HGNC:27424] | 26 | -1.90 |
| CIRBP | cold-inducible RNA-binding protein [Source:RefSeq peptide;Acc:NP_001029450] | 7 | -1.89 |
| COL22A1 | Uncharacterized protein [Source:UniProtKB/TrEMBL;Acc:E1BG60] | 14 | -1.89 |
| ATP6V0D2 | V-type proton ATPase subunit d 2 [Source:UniProtKB/Swiss-Prot;Acc:Q2KJB6] | 14 | 1.89 |
| PDGFRA | alpha-type platelet-derived growth factor receptor precursor [Source:RefSeq peptide;Acc:NP_001179274] | 6 | -1.88 |
| CXORF57 | Uncharacterized protein [Source:UniProtKB/TrEMBL;Acc:E1BEZ6] | X | 1.88 |
| TMEM97 | Transmembrane protein 97 [Source:UniProtKB/Swiss-Prot;Acc:Q3MHW7] | 19 | -1.87 |
| EIF2C2 | Bos taurus eukaryotic translation initiation factor 2C, 2 (EIF2C2), mRNA. [Source:RefSeq mRNA;Acc:NM_205794] | 14 | -1.87 |
| MFAP5 | Microfibrillar-associated protein 5 [Source:UniProtKB/Swiss-Prot;Acc:Q28022] | 5 | -1.87 |
| TXNIP | thioredoxin-interacting protein [Source:RefSeq peptide;Acc:NP_001095345] | 3 | 1.87 |
| INA | Alpha-internexin [Source:UniProtKB/Swiss-Prot;Acc:Q08DH7] | 26 | 1.86 |
| SLAIN2 | Bos taurus SLAIN motif family, member 2 (SLAIN2), mRNA. [Source:RefSeq mRNA;Acc:NM_001035405] | 6 | 1.86 |
| TKTL2 | Transketolase-like protein 2 [Source:UniProtKB/Swiss-Prot;Acc:Q2NKZ4] | 6 | 1.86 |
| ENPP4 | Ectonucleotide pyrophosphatase/phosphodiesterase family member 4 [Source:UniProtKB/Swiss-Prot;Acc:A2VDP5] | 23 | -1.86 |
| SFR1 | Uncharacterized protein [Source:UniProtKB/TrEMBL;Acc:G3MWJ1] | 26 | -1.86 |
| CPXM2 | inactive carboxypeptidase-like protein X2 precursor [Source:RefSeq peptide;Acc:NP_001192986] | 26 | -1.85 |
| ETNK1 | Uncharacterized protein [Source:UniProtKB/TrEMBL;Acc:F1N378] | 5 | 1.85 |
| DDIT3 | DNA damage-inducible transcript 3 protein [Source:UniProtKB/Swiss-Prot;Acc:Q0IIB6] | 5 | -1.85 |
| PPIG | Bos taurus peptidylprolyl isomerase G (cyclophilin G) (PPIG), mRNA. [Source:RefSeq mRNA;Acc:NM_001109807] | 2 | -1.84 |
| ENSBTAG00000018098 | Known pseudogene | X | 1.84 |
| CRYZ | Zeta-crystallin [Source:UniProtKB/Swiss-Prot;Acc:O97764] | 3 | 1.84 |
| MME | neprilysin [Source:RefSeq peptide;Acc:NP_001179813] | 1 | -1.84 |
| snoZ30 | Z30 small nucleolar RNA [Source:RFAM;Acc:RF00288] | 19 | 1.83 |
| NEFH | Uncharacterized protein [Source:UniProtKB/TrEMBL;Acc:F1MSQ6] | 17 | 1.83 |
| P2RX4 | P2X purinoceptor 4 [Source:UniProtKB/Swiss-Prot;Acc:Q5E9U1] | 17 | -1.82 |
| SUB1 | activated RNA polymerase II transcriptional coactivator p15 [Source:RefSeq peptide;Acc:NP_001098877] | 20 | -1.82 |
| CEP72 | Uncharacterized protein [Source:UniProtKB/TrEMBL;Acc:E1BPC1] | 20 | -1.82 |
| CYP2C87 | cytochrome P450, family 2, subfamily C, polypeptide 87 precursor [Source:RefSeq peptide;Acc:NP_001069895] | 26 | 1.81 |
| Unknown 2 | unknown | 16 | -1.81 |
| SUGP2 | Uncharacterized protein [Source:UniProtKB/TrEMBL;Acc:E1BE55] | 7 | -1.81 |
| CYP51A1 | Lanosterol 14-alpha demethylase [Source:UniProtKB/Swiss-Prot;Acc:Q4PJW3] | 4 | -1.81 |
| Unknown 3 | unknown | 15 | -1.81 |
| LOC100300759 | uncharacterized LOC100300759 | 21 | 1.80 |
| HSD3B | 3 beta-hydroxysteroid dehydrogenase/Delta 5-->4-isomerase 3-beta-hydroxy-Delta(5)-steroid dehydrogenase Steroid Delta-isomerase [Source:UniProtKB/Swiss-Prot;Acc:P14893] | 3 | 1.80 |
| ADH6 | alcohol dehydrogenase 6 [Source:RefSeq peptide;Acc:NP_001039522] | 6 | -1.80 |
| SH3BGR | SH3 domain binding glutamic acid-rich protein [Source:RefSeq peptide;Acc:NP_001230255] | 1 | 1.80 |
| SSBP2 | single-stranded DNA-binding protein 2 [Source:RefSeq peptide;Acc:NP_001030555] | 7 | -1.80 |
| SMARCA2 | probable global transcription activator SNF2L2 [Source:RefSeq peptide;Acc:NP_001092585] | 8 | -1.80 |
| FSTL1 | Follistatin-related protein 1 [Source:UniProtKB/Swiss-Prot;Acc:Q58D84] | 1 | -1.80 |
| TIMP2 | metalloproteinase inhibitor 2 precursor [Source:RefSeq peptide;Acc:NP_776897] | 19 | 1.80 |
| NEDD9 | NEDD9 protein; Uncharacterized protein [Source:UniProtKB/TrEMBL;Acc:A6QPB6] | 23 | 1.80 |
| TPPP3 | Tubulin polymerization-promoting protein family member 3 [Source:UniProtKB/Swiss-Prot;Acc:Q3ZCC8] | 18 | -1.79 |
| CLDN10 | Claudin-10 [Source:UniProtKB/Swiss-Prot;Acc:Q5E9L0] | 12 | 1.79 |
| EXOC3L4 | exocyst complex component 3-like protein 4 [Source:RefSeq peptide;Acc:NP_001192859] | 21 | 1.79 |
| Unknown 4 | unknown | 2 | 1.79 |
| CDK7 | cyclin-dependent kinase 7 [Source:RefSeq peptide;Acc:NP_001069183] | 20 | 1.79 |
| EFR3A | protein EFR3 homolog A [Source:RefSeq peptide;Acc:NP_001069515] | 14 | 1.79 |
| UNK | RING finger protein unkempt homolog [Source:RefSeq peptide;Acc:NP_001073722] | 19 | -1.79 |
| ETS1 | Bos taurus v-ets erythroblastosis virus E26 oncogene homolog 1 (avian) (ETS1), mRNA. [Source:RefSeq mRNA;Acc:NM_001099106] | 29 | -1.79 |
| HES6 | transcription cofactor HES-6 [Source:RefSeq peptide;Acc:NP_001069417] | 3 | 1.79 |
| ENSBTAG00000018481 | Uncharacterized protein [Source:UniProtKB/TrEMBL;Acc:E1BJP1] | 8 | 1.79 |
| BC153276 | Bos taurus cDNA clone IMAGE:8508304 | 18 | 1.78 |
| JAM2 | junctional adhesion molecule B precursor [Source:RefSeq peptide;Acc:NP_001077205] | 1 | -1.78 |
| RHOQ | rho-related GTP-binding protein RhoQ [Source:RefSeq peptide;Acc:NP_001192427] | 11 | 1.78 |
| MAGEB4 | melanoma antigen family B, 4 [Source:RefSeq peptide;Acc:NP_001070518] | X | 1.78 |
| CCDC82 | coiled-coil domain-containing protein 82 [Source:RefSeq peptide;Acc:NP_001039559] | 15 | 1.78 |
| COL18A1 | collagen alpha-1(XVIII) chain precursor [Source:RefSeq peptide;Acc:NP_001076857] | 1 | -1.78 |
| LOC527068 | aldo-keto reductase family 1 member C3 -like [Source:RefSeq peptide;Acc:NP_001030444] | 13 | 1.78 |
| CACNA1D | voltage-dependent L-type calcium channel subunit alpha-1D [Source:RefSeq peptide;Acc:NP_001179954] | 22 | -1.78 |
| PDPR | pyruvate dehydrogenase phosphatase regulatory subunit [Source:HGNC Symbol;Acc:HGNC:30264] | 18 | -1.78 |
| CRISP2 | cysteine-rich secretory protein 2 precursor [Source:RefSeq peptide;Acc:NP_001033178] | 23 | 1.78 |
| GPX8 | Probable glutathione peroxidase 8 [Source:UniProtKB/Swiss-Prot;Acc:Q2NL01] | 20 | -1.77 |
| ACTC1 | Actin, alpha cardiac muscle 1 [Source:UniProtKB/Swiss-Prot;Acc:Q3ZC07] | 10 | 1.77 |
| LOC508820 | melanoma-associated antigen 9-like [Source:RefSeq peptide;Acc:NP_001068841] | X | 1.77 |
| AP1S2 | AP-1 complex subunit sigma-2 [Source:UniProtKB/Swiss-Prot;Acc:Q3ZBS3] | X | -1.77 |
| RBMS2 | Bos taurus RNA binding motif, single stranded interacting protein 2 (RBMS2), mRNA. [Source:RefSeq mRNA;Acc:NM_001034365] | 5 | -1.77 |
| F2 | Prothrombin Activation peptide fragment 1 Activation peptide fragment 2 Thrombin light chain Thrombin heavy chain [Source:UniProtKB/Swiss-Prot;Acc:P00735] | 15 | -1.77 |
| CMBL | carboxymethylenebutenolidase homolog [Source:RefSeq peptide;Acc:NP_001179912] | 20 | 1.77 |
| LOC515042 | SCAN domain-containing protein 1-like [Source:RefSeq peptide;Acc:NP_001098840] | 2 | -1.77 |
| SCML4 | sex comb on midleg-like protein 4 [Source:RefSeq peptide;Acc:NP_001095654] | 9 | 1.77 |
| ENSBTAG00000046971 | Uncharacterized protein [Source:UniProtKB/TrEMBL;Acc:F1N0A2] | 11 | -1.77 |
| ZNHIT6 | Uncharacterized protein [Source:UniProtKB/TrEMBL;Acc:F1MYH0] | 3 | 1.77 |
| RAC2 | Ras-related C3 botulinum toxin substrate 2 [Source:UniProtKB/Swiss-Prot;Acc:Q9TU25] | 5 | 1.76 |
| H2AFZ | histone H2A.Z [Source:RefSeq peptide;Acc:NP_777234] | 6 | 1.76 |
| TPI1 | Triosephosphate isomerase [Source:UniProtKB/Swiss-Prot;Acc:Q5E956] | 5 | 1.76 |
| ALPL | Alkaline phosphatase, tissue-nonspecific isozyme [Source:UniProtKB/Swiss-Prot;Acc:P09487] | 2 | -1.76 |
| SPRED2 | sprouty-related, EVH1 domain containing 2 [Source:HGNC Symbol;Acc:HGNC:17722] | 11 | -1.76 |
| HES2 | transcription factor HES-2 isoform 1 [Source:RefSeq peptide;Acc:NP_001185934] | 16 | 1.76 |
| TNP1 | Spermatid nuclear transition protein 1 [Source:UniProtKB/Swiss-Prot;Acc:P17305] | 2 | 1.76 |
| ST3GAL6 | Bos taurus ST3 beta-galactoside alpha-2,3-sialyltransferase 6 (ST3GAL6), mRNA. [Source:RefSeq mRNA;Acc:NM_001002883] | 1 | -1.76 |
| EFNA1 | Ephrin-A1 Ephrin-A1, secreted form [Source:UniProtKB/Swiss-Prot;Acc:Q3ZC64] | 3 | -1.76 |
| SUV39H2 | Histone-lysine N-methyltransferase SUV39H2 [Source:UniProtKB/Swiss-Prot;Acc:Q32PH7] | 13 | 1.75 |
| MET | Hepatocyte growth factor receptor [Source:UniProtKB/Swiss-Prot;Acc:Q769I5] | 4 | -1.75 |
| UTP14A | U3 small nucleolar RNA-associated protein 14 homolog A [Source:UniProtKB/Swiss-Prot;Acc:Q3T0Q8] | X | -1.75 |
| FAM100B | Uncharacterized protein [Source:UniProtKB/TrEMBL;Acc:E1B848] | 19 | -1.75 |
| DENND1A | DENN domain-containing protein 1A [Source:RefSeq peptide;Acc:NP_001179943] | 11 | -1.75 |
| GFPT2 | Glucosamine--fructose-6-phosphate aminotransferase [isomerizing] 2 [Source:UniProtKB/Swiss-Prot;Acc:Q08DQ2] | 7 | 1.75 |
| RNF128 | E3 ubiquitin-protein ligase RNF128 precursor [Source:RefSeq peptide;Acc:NP_001069539] | X | 1.75 |
| ANTXR1 | anthrax toxin receptor 1 [Source:HGNC Symbol;Acc:HGNC:21014] | 11 | -1.74 |
| LOC539238 | ankyrin-2-like | 6 | -1.74 |
| SH3GL2 | endophilin-A1 [Source:RefSeq peptide;Acc:NP_001070308] | 8 | 1.74 |
| UTP14A | UTP14, U3 small nucleolar ribonucleoprotein, homolog A [Source:RefSeq peptide;Acc:NP_001093772] | 21 | -1.74 |
| EIF5 | eukaryotic translation initiation factor 5 [Source:RefSeq peptide;Acc:NP_001180152] | 21 | 1.74 |
| BC151663 | Bos taurus cDNA clone IMAGE:8459037. | 7 | -1.74 |
| PHLDA2 | pleckstrin homology-like domain family A member 2 [Source:RefSeq peptide;Acc:NP_001069989] | 29 | 1.74 |
| FHIT | Bos taurus fragile histidine triad gene (FHIT), mRNA. [Source:RefSeq mRNA;Acc:NM_001040646] | 22 | -1.73 |
| COL1A2 | Collagen alpha-2(I) chain [Source:UniProtKB/Swiss-Prot;Acc:P02465] | 4 | 1.73 |
| SPRY2 | Protein sprouty homolog 2 [Source:UniProtKB/Swiss-Prot;Acc:Q08E39] | 12 | -1.73 |
| MAGEB10 | Uncharacterized protein [Source:UniProtKB/TrEMBL;Acc:G3N1X1] | X | 1.73 |
| ATP9B | Probable phospholipid-transporting ATPase IIB [Source:UniProtKB/Swiss-Prot;Acc:A1A4J6] | 24 | -1.73 |
| IPPK | Uncharacterized protein [Source:UniProtKB/TrEMBL;Acc:E1BGX9] | 8 | 1.73 |
| TUBB2B | Tubulin beta-2B chain [Source:UniProtKB/Swiss-Prot;Acc:Q6B856] | 23 | 1.73 |
| GOLGA4 | Golgin subfamily A member 4 [Source:RefSeq peptide;Acc:NP_001179054] | 22 | -1.73 |
| GALT | galactose-1-phosphate uridylyltransferase [Source:RefSeq peptide;Acc:NP_001030213] | 8 | 1.72 |
| ENSBTAG00000007807 | Known pseudogene | 10 | 1.72 |
| U1 | U1 spliceosomal RNA [Source:RFAM;Acc:RF00003] ncRNA | 2 | -1.72 |
| BLVRB | Flavin reductase (NADPH) [Source:UniProtKB/Swiss-Prot;Acc:P52556] | 18 | 1.72 |
| ALDH4A1 | Delta-1-pyrroline-5-carboxylate dehydrogenase, mitochondrial [Source:UniProtKB/Swiss-Prot;Acc:A7YWE4] | 2 | -1.72 |
| DNAJC15 | dnaJ homolog subfamily C member 15 [Source:RefSeq peptide;Acc:NP_001073801] | 12 | 1.72 |
| GRAMD1A | Uncharacterized protein [Source:UniProtKB/TrEMBL;Acc:F6QVR0] | 18 | -1.71 |
| MARCKS | myristoylated alanine-rich C-kinase substrate [Source:RefSeq peptide;Acc:NP_001069744] | 9 | -1.71 |
| RHPN2 | rhophilin-2 [Source:RefSeq peptide;Acc:NP_001076939] | 18 | -1.71 |
| LAMB1 | laminin subunit beta-1 precursor [Source:RefSeq peptide;Acc:NP_001193448] | 4 | -1.71 |
| Unknown 4 | unknown | 12 | -1.71 |
| PDLIM4 | PDZ and LIM domain protein 4 [Source:UniProtKB/Swiss-Prot;Acc:Q3T005] | 7 | -1.71 |
| EVL | ena/VASP-like protein [Source:RefSeq peptide;Acc:NP_001179013] | 21 | -1.70 |
| RAB32 | ras-related protein Rab-32 [Source:RefSeq peptide;Acc:NP_001192879] | 9 | 1.70 |
| CALB1 | Calbindin [Source:UniProtKB/Swiss-Prot;Acc:P04467] | 14 | 1.70 |
| ISOC1 | Isochorismatase domain-containing protein 1 [Source:UniProtKB/Swiss-Prot;Acc:A6QLY4] | 7 | 1.70 |
| FABP5 | Fatty acid-binding protein, epidermal [Source:UniProtKB/TrEMBL;Acc:G3N269] | 14 | 1.70 |
| HN1 | Hematological and neurological expressed 1 protein [Source:UniProtKB/Swiss-Prot;Acc:Q3T0T5] | 19 | -1.70 |
| GSTO1 | glutathione-S-transferase omega 1 [Source:RefSeq peptide;Acc:NP_001068682] | 26 | 1.70 |
| RFC1 | Uncharacterized protein [Source:UniProtKB/TrEMBL;Acc:F1N4T1] | 6 | -1.70 |
| MYOF | myoferlin [Source:RefSeq peptide;Acc:NP_001180141] | 26 | -1.70 |
| ME1 | NADP-dependent malic enzyme [Source:RefSeq peptide;Acc:NP_001138325] | 9 | 1.70 |
| C9ORF95 | Uncharacterized protein [Source:UniProtKB/TrEMBL;Acc:F1N5H1] | 8 | 1.69 |
| NADK | NAD kinase [Source:RefSeq peptide;Acc:NP_001029617] | 16 | 1.69 |
| TMEM123 | transmembrane protein 123 | 15 | -1.69 |
| C19H17orf89 | chromosome 19 open reading frame, human C17orf89 | 19 | 1.69 |
| PDIA4 | protein disulfide-isomerase A4 precursor [Source:RefSeq peptide;Acc:NP_001039344] | 4 | -1.69 |
| PHLDA1 | Bos taurus pleckstrin homology-like domain, family A, member 1 (PHLDA1), mRNA. [Source:RefSeq mRNA;Acc:NM_001105631] | 5 | -1.69 |
| RIP5 | RAB5-interacting protein [Source:RefSeq peptide;Acc:NP_001029593] | 13 | 1.69 |
| CHORDC1 | Cysteine and histidine-rich domain-containing protein 1 [Source:UniProtKB/Swiss-Prot;Acc:Q29RL2] | 29 | 1.69 |
| HBP1 | HMG box-containing protein 1 [Source:RefSeq peptide;Acc:NP_001039661] | 4 | -1.69 |
| SREBF2 | sterol regulatory element-binding protein 2 [Source:RefSeq peptide;Acc:NP_001192529] | 5 | -1.69 |
| ISCA1 | Iron-sulfur cluster assembly 1 homolog, mitochondrial [Source:UniProtKB/Swiss-Prot;Acc:Q3SZG8] | 8 | 1.68 |
| PSAT1 | phosphoserine aminotransferase [Source:RefSeq peptide;Acc:NP_001095620] | 8 | 1.68 |
| PTPRK | receptor-type tyrosine-protein phosphatase kappa precursor [Source:RefSeq peptide;Acc:NP_001178466] | 9 | -1.68 |
| GCH1 | Uncharacterized protein [Source:UniProtKB/TrEMBL;Acc:F1MZ14] | 10 | 1.68 |
| CNRIP1 | CB1 cannabinoid receptor-interacting protein 1 [Source:UniProtKB/Swiss-Prot;Acc:Q17QM9] | 11 | -1.68 |
| CCNE1 | G1/S-specific cyclin-E1 [Source:RefSeq peptide;Acc:NP_001179705] | 18 | 1.68 |
| GPR125 | Uncharacterized protein [Source:UniProtKB/TrEMBL;Acc:F1MK80] | 6 | -1.68 |
| ARHGEF2 | rho guanine nucleotide exchange factor 2 [Source:RefSeq peptide;Acc:NP_001092351] | 3 | -1.68 |
| DNASE1 | Deoxyribonuclease-1 [Source:UniProtKB/Swiss-Prot;Acc:P00639] | 25 | -1.68 |
| JMJD6 | Bifunctional arginine demethylase and lysyl-hydroxylase JMJD6 [Source:UniProtKB/Swiss-Prot;Acc:Q58DS6] | 19 | 1.67 |
| DHCR7 | 7-dehydrocholesterol reductase [Source:RefSeq peptide;Acc:NP_001014927] | 29 | -1.67 |
| SUPT4H1 | Transcription elongation factor SPT4 [Source:UniProtKB/Swiss-Prot;Acc:Q3SYX6] | 19 | 1.67 |
| SLC25A30 | kidney mitochondrial carrier protein 1 [Source:RefSeq peptide;Acc:NP_001092365] | 12 | -1.67 |
| TMEM80 | Transmembrane protein 80 [Source:UniProtKB/Swiss-Prot;Acc:A1A4P6] | 29 | -1.67 |
| MTUS1 | Microtubule-associated tumor suppressor 1 homolog [Source:UniProtKB/Swiss-Prot;Acc:Q17QT2] | 27 | -1.67 |
| HOXD4 | homeobox protein Hox-D4 [Source:RefSeq peptide;Acc:NP_001094557] | 2 | -1.67 |
| LOC100337293 | ankyrin repeat domain-containing protein 26-like | 13 | -1.67 |
| ITGA5 | integrin alpha-5 [Source:RefSeq peptide;Acc:NP_001159972] | 5 | -1.67 |
| TPMT | Thiopurine S-methyltransferase [Source:UniProtKB/Swiss-Prot;Acc:Q17QQ2] | 23 | 1.67 |
| Unknown 5 | unknown | 24 | -1.67 |
| ID3 | DNA-binding protein inhibitor ID-3 [Source:UniProtKB/Swiss-Prot;Acc:Q5E981] | 2 | -1.67 |
| CD47 | leukocyte surface antigen CD47 precursor [Source:RefSeq peptide;Acc:NP_777133] | 1 | 1.67 |
| GNG4 | guanine nucleotide-binding protein G(I)/G(S)/G(O) subunit gamma-4 [Source:RefSeq peptide;Acc:NP_001096794] | 28 | -1.67 |
| NEBL | nebulette [Source:HGNC Symbol;Acc:HGNC:16932] | 13 | -1.67 |
| TMEM206 | Transmembrane protein 206 [Source:UniProtKB/Swiss-Prot;Acc:Q2KHV2] | 16 | -1.67 |
| ANKH | progressive ankylosis protein homolog [Source:RefSeq peptide;Acc:NP_001103263] | 20 | -1.66 |
| PDLIM7 | PDZ and LIM domain protein 7 [Source:UniProtKB/Swiss-Prot;Acc:Q3SX40] | 7 | 1.66 |
| MGC142781 | carnitine O-acetyltransferase [Source:RefSeq peptide;Acc:NP_001069055] | 11 | 1.66 |
| BCL2L2 | Bcl-2-like protein 2 [Source:UniProtKB/Swiss-Prot;Acc:Q1RMX3] | 10 | -1.66 |
| Unknown 6 | unknown | 29 | -1.66 |
| C3H1orf146 | Uncharacterized protein C1orf146 homolog [Source:UniProtKB/Swiss-Prot;Acc:Q2TA05] | 3 | 1.66 |
| BBX | Bos taurus bobby sox homolog (Drosophila) (BBX), mRNA. [Source:RefSeq mRNA;Acc:NM_001192759] | 1 | -1.66 |
| UNG | uracil-DNA glycosylase [Source:RefSeq peptide;Acc:NP_001068981] | 17 | -1.66 |
| Unknown 7 | unknown | 5 | -1.66 |
| TMEM139 | transmembrane protein 139 [Source:RefSeq peptide;Acc:NP_001137344] | 4 | 1.66 |
| PIGV | GPI mannosyltransferase 2 [Source:RefSeq peptide;Acc:NP_001179434] | 2 | -1.66 |
| TRIM25 | E3 ubiquitin/ISG15 ligase TRIM25 [Source:RefSeq peptide;Acc:NP_001093806] | 19 | 1.66 |
| ENSBTAG00000034761 | Uncharacterized protein [Source:UniProtKB/TrEMBL;Acc:G5E607] | 17 | 1.66 |
| RNF19B | E3 ubiquitin-protein ligase RNF19B [Source:RefSeq peptide;Acc:NP_001192591] | 2 | 1.66 |
| CALCOCO2 | Calcium-binding and coiled-coil domain-containing protein 2 [Source:UniProtKB/Swiss-Prot;Acc:O18737] | 19 | 1.66 |
| ENSBTAG00000038327 | Uncharacterized protein [Source:UniProtKB/TrEMBL;Acc:F1ME51] | 10 | 1.66 |
| CRISPLD2 | Cysteine-rich secretory protein LCCL domain-containing 2 [Source:UniProtKB/Swiss-Prot;Acc:A6QLZ7] | 18 | 1.66 |
| SLC20A1 | sodium-dependent phosphate transporter 1 [Source:RefSeq peptide;Acc:NP_001178114] | 11 | -1.65 |
| DEM1 | probable exonuclease V [Source:RefSeq peptide;Acc:NP_001075077] | 3 | 1.65 |
| NGFR | tumor necrosis factor receptor superfamily member 16 precursor [Source:RefSeq peptide;Acc:NP_001095948] | 19 | -1.65 |
| RECS1 | transmembrane BAX inhibitor motif-containing protein 1 [Source:RefSeq peptide;Acc:NP_991367] | 2 | -1.65 |
| APBB3 | amyloid beta A4 precursor protein-binding family B member 3 [Source:RefSeq peptide;Acc:NP_001069335] | 7 | -1.65 |
| HYOU1 | hypoxia up-regulated protein 1 precursor [Source:RefSeq peptide;Acc:NP_001193839] | 15 | -1.65 |
| LOC787476 | Uncharacterized protein [Source:UniProtKB/TrEMBL;Acc:G5E5D5] | X | 1.65 |
| CGN | cingulin [Source:RefSeq peptide;Acc:NP_001179715] | 3 | -1.65 |
| SLC39A14 | Zinc transporter ZIP14 [Source:UniProtKB/Swiss-Prot;Acc:A5D7L5] | 8 | -1.65 |
| LOC540312 | protein DDX26B-like | X | 1.65 |
| ECHDC1 | enoyl-CoA hydratase domain-containing protein 1 [Source:RefSeq peptide;Acc:NP_001030492] | 9 | 1.65 |
| LSR | lipolysis-stimulated lipoprotein receptor [Source:RefSeq peptide;Acc:NP_001076863] | 18 | -1.64 |
| NDRG1 | protein NDRG1 [Source:RefSeq peptide;Acc:NP_001030181] | 14 | 1.64 |
| CROCC | Uncharacterized protein [Source:UniProtKB/TrEMBL;Acc:E1BBS9] | 2 | -1.64 |
| CALML5 | calmodulin-like 5 [Source:RefSeq peptide;Acc:NP_001091518] | 13 | -1.64 |
| Unknown 8 | unknown | 2 | -1.64 |
| FOS | Proto-oncogene c-Fos [Source:UniProtKB/Swiss-Prot;Acc:O77628] | 10 | -1.64 |
| RBM3 | Uncharacterized protein [Source:UniProtKB/TrEMBL;Acc:F6RBQ9] | X | -1.64 |
| SNCA | Alpha-synuclein [Source:UniProtKB/Swiss-Prot;Acc:Q3T0G8] | 6 | -1.64 |
| CPEB3 | cytoplasmic polyadenylation element-binding protein 3 [Source:RefSeq peptide;Acc:NP_001180157] | 26 | -1.64 |
| LOC101907314 | Uncharacterized protein [Source:UniProtKB/TrEMBL;Acc:G3MZD8] | 23 | -1.64 |
| GLI1 | zinc finger protein GLI1 [Source:RefSeq peptide;Acc:NP_001092470] | 5 | -1.64 |
| TXN | Thioredoxin [Source:UniProtKB/Swiss-Prot;Acc:O97680] | 8 | 1.64 |
| FAM65C | Uncharacterized protein [Source:UniProtKB/TrEMBL;Acc:F1MZ75] | 13 | 1.64 |
| Unknown 9 | unknown | 9 | -1.64 |
| BCAS3 | breast carcinoma amplified sequence 3 [Source:HGNC Symbol;Acc:HGNC:14347] | 19 | -1.63 |
| C29H11orf73 | Protein Hikeshi [Source:UniProtKB/Swiss-Prot;Acc:Q56JY0] | 29 | 1.63 |
| IL32 | interleukin 32 | 25 | -1.63 |
| PTPRJ | Uncharacterized protein [Source:UniProtKB/TrEMBL;Acc:F1MM04] | 15 | -1.63 |
| snoR38 | Small nucleolar RNA R38 [Source:RFAM;Acc:RF00213] | 19 | -1.63 |
| CDC26 | Anaphase-promoting complex subunit CDC26 [Source:UniProtKB/Swiss-Prot;Acc:Q3SZT7] | 8 | 1.63 |
| RNASEK | Ribonuclease kappa [Source:UniProtKB/Swiss-Prot;Acc:Q3ZC23] | 19 | 1.63 |
| ENSBTAG00000030454 | Uncharacterized protein [Source:UniProtKB/TrEMBL;Acc:E1BKQ7] | 18 | 1.63 |
| UGCG | Bos taurus UDP-glucose ceramide glucosyltransferase (UGCG), mRNA. [Source:RefSeq mRNA;Acc:NM_001076850] | 8 | -1.63 |
| NEDD4 | neural precursor cell expressed, developmentally down-regulated 4, E3 ubiquitin protein ligase [Source:HGNC Symbol;Acc:HGNC:7727] | 10 | -1.62 |
| HNRPDL | heterogeneous nuclear ribonucleoprotein D-like [Source:RefSeq peptide;Acc:NP_001077194] | 6 | -1.62 |
| NIPAL3 | NIPA-like domain containing 3 [Source:HGNC Symbol;Acc:HGNC:25233] | 2 | -1.62 |
| ASZ1 | Ankyrin repeat, SAM and basic leucine zipper domain-containing protein 1 [Source:UniProtKB/Swiss-Prot;Acc:Q8WMX8] | 4 | 1.62 |
| MYH1 | Myosin-1 [Source:UniProtKB/Swiss-Prot;Acc:Q9BE40] | 19 | -1.62 |
| MBTD1 | mbt domain containing 1 [Source:HGNC Symbol;Acc:HGNC:19866] | 19 | -1.62 |
| HSPA8 | Heat shock cognate 71 kDa protein [Source:UniProtKB/Swiss-Prot;Acc:P19120] | 15 | 1.62 |
| STOM | stomatin [Source:HGNC Symbol;Acc:HGNC:3383] | 8 | -1.62 |
| C20H5orf41 | chromosome 20 open reading frame, human C5orf41 | 20 | -1.62 |
| LOC100848555 | X antigen family member 5-like | X | 1.62 |
| KPNA4 | importin subunit alpha-4 [Source:RefSeq peptide;Acc:NP_001152788] | 1 | 1.62 |
| LOXL2 | Lysyl oxidase homolog 2 [Source:UniProtKB/Swiss-Prot;Acc:A6H737] | 8 | -1.62 |
| ARMCX6 | protein ARMCX6 [Source:RefSeq peptide;Acc:NP_001071593] | X | 1.62 |
| EIF3J | Eukaryotic translation initiation factor 3 subunit J [Source:UniProtKB/Swiss-Prot;Acc:Q0VCU8] | 10 | 1.62 |
| TAF7 | transcription initiation factor TFIID subunit 7 [Source:RefSeq peptide;Acc:NP_001039493] | 7 | -1.62 |
| CDC45L | cell division control protein 45 homolog [Source:RefSeq peptide;Acc:NP_001019661] | 17 | -1.62 |
| TMEM106C | Transmembrane protein 106C [Source:UniProtKB/Swiss-Prot;Acc:Q3T144] | 5 | 1.61 |
| SNORA17 | Small nucleolar RNA SNORA17 [Source:RFAM;Acc:RF00560] | 11 | -1.61 |
| MGC140151 | protein BEX4 [Source:RefSeq peptide;Acc:NP_001103566] | X | 1.61 |
| LRRFIP1 | leucine-rich repeat flightless-interacting protein 1 [Source:RefSeq peptide;Acc:NP_001095778] | 3 | -1.61 |
| FUT5 | galactoside 3(4)-L-fucosyltransferase [Source:RefSeq peptide;Acc:NP_789821] | 7 | -1.61 |
| ITGB2 | Integrin beta-2 [Source:UniProtKB/Swiss-Prot;Acc:P32592] | 1 | 1.61 |
| NFE2L2 | nuclear factor erythroid 2-related factor 2 [Source:RefSeq peptide;Acc:NP_001011678] | 2 | 1.61 |
| H2B | histone H2B type 1-N [Source:RefSeq peptide;Acc:NP_001075211] | 23 | 1.61 |
| LSM12 | Protein LSM12 homolog [Source:UniProtKB/Swiss-Prot;Acc:Q0VCF9] | 19 | 1.61 |
| PTP4A1 | protein tyrosine phosphatase type IVA 1 [Source:RefSeq peptide;Acc:NP_001193053] | 9 | 1.61 |
| ZNF710 | zinc finger protein 710 [Source:HGNC Symbol;Acc:HGNC:25352] | 21 | -1.61 |
| SPG11 | Uncharacterized protein [Source:UniProtKB/TrEMBL;Acc:F1MEB4] | 10 | -1.61 |
| ZNRF1 | E3 ubiquitin-protein ligase ZNRF1 [Source:UniProtKB/Swiss-Prot;Acc:F1MM41] | 18 | -1.61 |
| CLTA | Bos taurus clathrin, light chain A (CLTA), mRNA. [Source:RefSeq mRNA;Acc:NM_174022] | 8 | -1.61 |
| DSC2 | desmocollin-2 precursor [Source:RefSeq peptide;Acc:NP_001159998] | 24 | -1.61 |
| GPR107 | Bos taurus G protein-coupled receptor 107 (GPR107), mRNA. [Source:RefSeq mRNA;Acc:NM_001099164] | 11 | -1.61 |
| SLC26A6 | solute carrier family 26 member 6 [Source:RefSeq peptide;Acc:NP_001070320] | 22 | -1.61 |
| GFPT1 | Bos taurus glutamine--fructose-6-phosphate transaminase 1 (GFPT1), mRNA. [Source:RefSeq mRNA;Acc:NM_001109961] | 11 | -1.60 |
| EIF1AY | eukaryotic translation initiation factor 1A, Y-linked [Source:RefSeq peptide;Acc:NP_001139229] | X | 1.60 |
| NQO1 | NAD(P)H dehydrogenase [Source:RefSeq peptide;Acc:NP_001029707] | 18 | 1.60 |
| ALDH2 | Aldehyde dehydrogenase, mitochondrial [Source:UniProtKB/Swiss-Prot;Acc:P20000] | 17 | -1.60 |
| TRADD | Tumor necrosis factor receptor type 1-associated DEATH domain protein [Source:UniProtKB/Swiss-Prot;Acc:Q2KI74] | 18 | 1.60 |
| LOC101909651 | Uncharacterized protein [Source:UniProtKB/TrEMBL;Acc:G8JKV3] | 16 | 1.60 |
| THEX1 | 3'-5' exoribonuclease 1 [Source:RefSeq peptide;Acc:NP_001033281] | 27 | 1.60 |
| PIM2 | serine/threonine-protein kinase pim-2 [Source:RefSeq peptide;Acc:NP_001193307] | X | -1.60 |
| Unknown 10 | unknown | 13 | -1.60 |
| ATP6V1G3 | Uncharacterized protein [Source:UniProtKB/TrEMBL;Acc:E1BMV6] | 16 | 1.60 |
| ES1 | ES1 protein homolog, mitochondrial [Source:RefSeq peptide;Acc:NP_001029635] | 1 | -1.60 |
| GSDMC | gasdermin-C [Source:RefSeq peptide;Acc:NP_001039469] | 14 | 1.60 |
| HPGD | 15-hydroxyprostaglandin dehydrogenase [Source:RefSeq peptide;Acc:NP_001029591] | 8 | 1.60 |
| MKNK1 | MAP kinase-interacting serine/threonine-protein kinase 1 [Source:RefSeq peptide;Acc:NP_001030435] | 3 | 1.60 |
| SLC43A3 | Solute carrier family 43 member 3 [Source:UniProtKB/Swiss-Prot;Acc:Q1JPD8] | 15 | -1.60 |
| SOCS1 | Uncharacterized protein [Source:UniProtKB/TrEMBL;Acc:F1MSN0] | 25 | 1.59 |
| GPI | Glucose-6-phosphate isomerase [Source:UniProtKB/Swiss-Prot;Acc:Q3ZBD7] | 18 | 1.59 |
| ETF1 | Eukaryotic peptide chain release factor subunit 1 [Source:UniProtKB/Swiss-Prot;Acc:Q0VCX5] | 7 | 1.59 |
| LCP1 | plastin-2 [Source:RefSeq peptide;Acc:NP_001029892] | 12 | 1.59 |
| CAV1 | Caveolin-1 [Source:UniProtKB/Swiss-Prot;Acc:P79132] | 4 | -1.59 |
| DNM1L | Dynamin-1-like protein [Source:UniProtKB/Swiss-Prot;Acc:Q2KIA5] | 5 | 1.59 |
| ENSBTAG00000020953 | Novel pseudogene | 16 | -1.59 |
| ACAT1 | Acetyl-CoA acetyltransferase, mitochondrial [Source:UniProtKB/Swiss-Prot;Acc:Q29RZ0] | 15 | -1.59 |
| CTSO | Uncharacterized protein [Source:UniProtKB/TrEMBL;Acc:E1BPI9] | 17 | -1.59 |
| LOC780876 | mitotic checkpoint component Mad2 [Source:RefSeq peptide;Acc:NP_001073264] | 6 | 1.59 |
| ATP6V1B1 | V-type proton ATPase subunit B, kidney isoform [Source:RefSeq peptide;Acc:NP_788827] | 11 | 1.59 |
| ACTB | Uncharacterized protein [Source:UniProtKB/TrEMBL;Acc:F1MKC4] | 11 | -1.58 |
| STXBP1 | Syntaxin-binding protein 1 [Source:UniProtKB/Swiss-Prot;Acc:P61763] | 11 | -1.58 |
| IL6 | Interleukin-6 [Source:UniProtKB/Swiss-Prot;Acc:P26892] | 4 | 1.58 |
| ABCE1 | ATP-binding cassette sub-family E member 1 [Source:RefSeq peptide;Acc:NP_001077154] | 17 | 1.58 |
| TMOD1 | Tropomodulin-1 [Source:UniProtKB/Swiss-Prot;Acc:A0JNC0] | 8 | -1.58 |
| PEX10 | peroxisome biogenesis factor 10 [Source:RefSeq peptide;Acc:NP_001069801] | 16 | -1.58 |
| SOD1 | Superoxide dismutase [Cu-Zn] [Source:UniProtKB/Swiss-Prot;Acc:P00442] | 1 | 1.58 |
| CXADR | Bos taurus coxsackie virus and adenovirus receptor (CXADR), mRNA. [Source:RefSeq mRNA;Acc:NM_174298] | 1 | -1.58 |
| NID1 | Uncharacterized protein [Source:UniProtKB/TrEMBL;Acc:F1MWN3] | 28 | -1.58 |
| MVK | mevalonate kinase [Source:RefSeq peptide;Acc:NP_001015528] | 17 | -1.58 |
| Unknown 11 | unknown | X | -1.58 |
| CCNE2 | G1/S-specific cyclin-E2 [Source:UniProtKB/Swiss-Prot;Acc:Q5E9K7] | 14 | 1.58 |
| CELSR1 | Uncharacterized protein [Source:UniProtKB/TrEMBL;Acc:F1N6Y9] | 5 | -1.57 |
| LRP1 | Uncharacterized protein [Source:UniProtKB/TrEMBL;Acc:E1BGJ0] | 5 | -1.57 |
| MYLK | myosin light chain kinase, smooth muscle [Source:RefSeq peptide;Acc:NP_788809] | 1 | 1.57 |
| STRN | striatin [Source:RefSeq peptide;Acc:NP_001193033] | 11 | 1.57 |
| MACF1 | Uncharacterized protein [Source:UniProtKB/TrEMBL;Acc:F1N6H4] | 3 | -1.57 |
| LCK | tyrosine-protein kinase Lck [Source:RefSeq peptide;Acc:NP_001029506] | 2 | 1.57 |
| TNRC6A | trinucleotide repeat containing 6A [Source:HGNC Symbol;Acc:HGNC:11969] | 25 | -1.57 |
| TUBB6 | Tubulin beta-6 chain [Source:UniProtKB/TrEMBL;Acc:G3X7R8] | 24 | 1.57 |
| EEF2 | Elongation factor 2 [Source:UniProtKB/Swiss-Prot;Acc:Q3SYU2] | 7 | -1.57 |
| TMEM9B | transmembrane protein 9B precursor [Source:RefSeq peptide;Acc:NP_001029679] | 15 | 1.57 |
| Unknown 12 | unknown | 2 | -1.57 |
| ASRGL1 | L-asparaginase [Source:UniProtKB/Swiss-Prot;Acc:Q32LE5] | 29 | 1.57 |
| ENSBTAG00000030507 | Ferritin [Source:UniProtKB/TrEMBL;Acc:E1B888] | X | 1.57 |
| C1H3orf70 | chromosome 3 open reading frame 70 [Source:HGNC Symbol;Acc:HGNC:33731] | 1 | -1.57 |
| PPP1CC | Serine/threonine-protein phosphatase PP1-gamma catalytic subunit [Source:UniProtKB/Swiss-Prot;Acc:P61287] | 17 | 1.57 |
| NEFL | Neurofilament light polypeptide [Source:UniProtKB/Swiss-Prot;Acc:P02548] | 8 | 1.57 |
| CDC37L1 | Hsp90 co-chaperone Cdc37-like 1 [Source:UniProtKB/Swiss-Prot;Acc:A6H754] | 8 | 1.57 |
| UBE2K | Ubiquitin-conjugating enzyme E2 K [Source:UniProtKB/Swiss-Prot;Acc:P61085] | 6 | 1.57 |
| SLC10A1 | sodium/bile acid cotransporter [Source:RefSeq peptide;Acc:NP_001039804] | 10 | -1.57 |
| GLB1 | beta-galactosidase precursor [Source:RefSeq peptide;Acc:NP_001030215] | 22 | -1.56 |
| ACSS2 | acetyl-coenzyme A synthetase, cytoplasmic [Source:RefSeq peptide;Acc:NP_001098809] | 13 | -1.56 |
| PDZRN3 | Uncharacterized protein [Source:UniProtKB/TrEMBL;Acc:F1MRX7] | 22 | 1.56 |
| SYNE2 | nesprin-2 [Source:RefSeq peptide;Acc:NP_001193515] | 10 | -1.56 |
| KCTD12 | potassium channel tetramerization domain containing 12 [Source:HGNC Symbol;Acc:HGNC:14678] | 12 | 1.56 |
| SYBU | syntabulin [Source:RefSeq peptide;Acc:NP_001069979] | 14 | 1.56 |
| Unknnown 13 | unknown |  | 1.56 |
| SRN | Seminal ribonuclease [Source:UniProtKB/Swiss-Prot;Acc:P00669] | 10 | 1.56 |
| TACSTD2 | Uncharacterized protein [Source:UniProtKB/TrEMBL;Acc:F1MSN2] | 3 | -1.56 |
| ENSBTAG00000033558 | Uncharacterized protein [Source:UniProtKB/TrEMBL;Acc:G3X806] | 17 | 1.56 |
| FLT3 | Uncharacterized protein [Source:UniProtKB/TrEMBL;Acc:F1MRU0] | 12 | -1.56 |
| bta-let-7d | bta-let-7d [Source:miRBase;Acc:MI0005026] | 8 | -1.56 |
| MAD2L1 | mitotic spindle assembly checkpoint protein MAD2A [Source:RefSeq peptide;Acc:NP_001178442] | 6 | 1.56 |
| FES | tyrosine-protein kinase Fes/Fps [Source:RefSeq peptide;Acc:NP_001027471] | 21 | -1.56 |
| GUSB | beta-glucuronidase precursor [Source:RefSeq peptide;Acc:NP_001076905] | 25 | 1.56 |
| LOC100337261 | butyrophilin, subfamily 1, member A1-like | 23 | 1.56 |
| SRXN1 | sulfiredoxin-1 [Source:RefSeq peptide;Acc:NP_001192917] | 13 | 1.56 |
| P4HA3 | Prolyl 4-hydroxylase subunit alpha-3 [Source:UniProtKB/Swiss-Prot;Acc:Q75UG4] | 15 | -1.56 |
| THEM4 | thioesterase superfamily member 4 [Source:RefSeq peptide;Acc:NP_001073837] | 3 | 1.56 |
| SLC9A9 | sodium/hydrogen exchanger 9 [Source:RefSeq peptide;Acc:NP_001069536] | 1 | -1.56 |
| CTNND2 | Uncharacterized protein [Source:UniProtKB/TrEMBL;Acc:F1MM16] | 20 | -1.56 |
| ANKRD1 | Ankyrin repeat domain-containing protein 1 [Source:UniProtKB/Swiss-Prot;Acc:Q3ZBX7] | 26 | -1.56 |
| KBTBD8 | Bos taurus kelch repeat and BTB (POZ) domain containing 8 (KBTBD8), mRNA. [Source:RefSeq mRNA;Acc:NM_001192696] | 22 | 1.56 |
| MTMR11 | myotubularin-related protein 11 [Source:RefSeq peptide;Acc:NP_001030202] | 3 | -1.56 |
| MEG3 | maternally expressed 3 (non-protein coding) | 21 | -1.56 |
| SEC24D | Uncharacterized protein [Source:UniProtKB/TrEMBL;Acc:F1MV07] | 6 | -1.56 |
| FBXL20 | F-box/LRR-repeat protein 20 [Source:UniProtKB/Swiss-Prot;Acc:Q58DG6] | 19 | -1.56 |
| SYNCRIP | Uncharacterized protein [Source:UniProtKB/TrEMBL;Acc:F1MCT8] | 9 | 1.56 |
| XIST | X (inactive)-specific transcript | X | -1.56 |
| ABHD4 | Abhydrolase domain-containing protein 4 [Source:UniProtKB/Swiss-Prot;Acc:Q5EA59] | 10 | 1.56 |
| SERP1 | Stress-associated endoplasmic reticulum protein 1 [Source:UniProtKB/Swiss-Prot;Acc:Q3ZBR1] | 1 | 1.56 |
| LOC783399 | Uncharacterized protein [Source:UniProtKB/TrEMBL;Acc:G3MZU3] | 8 | 1.56 |
| AHSA1 | activator of 90 kDa heat shock protein ATPase homolog 1 [Source:RefSeq peptide;Acc:NP_001029838] | 10 | 1.55 |
| GCLM | Glutamate--cysteine ligase regulatory subunit [Source:UniProtKB/Swiss-Prot;Acc:Q2T9Y6] | 3 | 1.55 |
| NTAN1 | protein N-terminal asparagine amidohydrolase [Source:RefSeq peptide;Acc:NP_001193073] | 25 | 1.55 |
| Unknown 14 | unknown | 10 | -1.55 |
| DENND5B | DENN/MADD domain containing 5B [Source:HGNC Symbol;Acc:HGNC:28338] | 5 | -1.55 |
| F3 | Tissue factor [Source:UniProtKB/Swiss-Prot;Acc:P30931] | 3 | 1.55 |
| ITFG1 | T-cell immunomodulatory protein precursor [Source:RefSeq peptide;Acc:NP_001033621] | 18 | -1.55 |
| REPIN1 | Replication initiator 1 [Source:UniProtKB/Swiss-Prot;Acc:Q0VCC5] | 4 | 1.55 |
| OTUD1 | Uncharacterized protein [Source:UniProtKB/TrEMBL;Acc:G3N2I1] | 13 | -1.55 |
| ADCY2 | Uncharacterized protein [Source:UniProtKB/TrEMBL;Acc:F1MJV4] | 20 | -1.55 |
| CA13 | Uncharacterized protein [Source:UniProtKB/TrEMBL;Acc:F1MIP9] | 14 | -1.55 |
| ADCK3 | Chaperone activity of bc1 complex-like, mitochondrial [Source:UniProtKB/Swiss-Prot;Acc:Q29RI0] | 16 | -1.55 |
| PDE9A | Uncharacterized protein [Source:UniProtKB/TrEMBL;Acc:F1MH30] | 1 | -1.55 |
| TMEM65 | Transmembrane protein 65 [Source:UniProtKB/Swiss-Prot;Acc:Q0VCH8] | 14 | 1.55 |
| CHD9 | chromodomain helicase DNA binding protein 9 [Source:HGNC Symbol;Acc:HGNC:25701] | 18 | -1.55 |
| BHLHE40 | Class E basic helix-loop-helix protein 40 [Source:UniProtKB/Swiss-Prot;Acc:Q5EA15] | 22 | -1.55 |
| DEFB | beta-defensin precursor [Source:RefSeq peptide;Acc:NP_001071601] | 27 | -1.55 |
| Unknown 15 | unknown | 13 | -1.55 |
| MAP6D1 | MAP6 domain-containing protein 1 [Source:UniProtKB/Swiss-Prot;Acc:Q0P591] | 1 | -1.54 |
| SLC16A12 | solute carrier family 16, member 12 [Source:HGNC Symbol;Acc:HGNC:23094] | 26 | -1.54 |
| JRKL | Uncharacterized protein [Source:UniProtKB/TrEMBL;Acc:F1MTE4] | 15 | -1.54 |
| NPC1 | Niemann-Pick C1 protein precursor [Source:RefSeq peptide;Acc:NP_777183] | 24 | -1.54 |
| RRM2 | ribonucleoside-diphosphate reductase subunit M2 isoform 1 [Source:RefSeq peptide;Acc:NP_001231110] | 11 | 1.54 |
| GCAT | 2-amino-3-ketobutyrate coenzyme A ligase, mitochondrial [Source:UniProtKB/Swiss-Prot;Acc:Q0P5L8] | 5 | -1.54 |
| CSRP2 | Cysteine and glycine-rich protein 2 [Source:UniProtKB/Swiss-Prot;Acc:Q32LE9] | 5 | 1.54 |
| MIB1 | Bos taurus mindbomb homolog 1 (Drosophila) (MIB1), mRNA. [Source:RefSeq mRNA;Acc:NM_001206030] | 24 | -1.54 |
| HSD17B7 | 3-keto-steroid reductase [Source:RefSeq peptide;Acc:NP_001076844] | 3 | -1.54 |
| FRMD5 | FERM domain containing 5 [Source:HGNC Symbol;Acc:HGNC:28214] | 21 | -1.54 |
| AZIN1 | antizyme inhibitor 1 [Source:RefSeq peptide;Acc:NP_001076080] | 14 | 1.54 |
| ABCB8 | ATP-binding cassette sub-family B member 8, mitochondrial [Source:RefSeq peptide;Acc:NP_001068994] | 4 | -1.54 |
| SNX18 | sorting nexin-18 [Source:RefSeq peptide;Acc:NP_001077200] | 20 | 1.54 |
| CYSTM1 | Cysteine-rich and transmembrane domain-containing protein 1 [Source:UniProtKB/Swiss-Prot;Acc:Q32LK2] | 7 | 1.54 |
| TRIM32 | E3 ubiquitin-protein ligase TRIM32 [Source:RefSeq peptide;Acc:NP_001069292] | 8 | 1.54 |
| ZNF292 | zinc finger protein 292 [Source:RefSeq peptide;Acc:NP_001179779] | 9 | -1.54 |
| DNAJB1 | DnaJ homolog subfamily B member 1 [Source:UniProtKB/Swiss-Prot;Acc:Q3MI00] | 7 | 1.54 |
| HINT3 | Histidine triad nucleotide-binding protein 3 [Source:UniProtKB/Swiss-Prot;Acc:Q2YDJ4] | 9 | 1.54 |
| NIN | ninein (GSK3B interacting protein) [Source:HGNC Symbol;Acc:HGNC:14906] | 10 | -1.54 |
| SQLE | squalene monooxygenase [Source:RefSeq peptide;Acc:NP_001091530] | 14 | -1.54 |
| SLC25A4 | ADP/ATP translocase 1 [Source:UniProtKB/Swiss-Prot;Acc:P02722] | 27 | 1.53 |
| TMEM45A | transmembrane protein 45A [Source:RefSeq peptide;Acc:NP_001068873] | 1 | -1.53 |
| TGFBR3 | Uncharacterized protein [Source:UniProtKB/TrEMBL;Acc:E1B9H5] | 3 | -1.53 |
| BCOR | BCL-6 corepressor [Source:RefSeq peptide;Acc:NP_001178473] | X | -1.53 |
| AFF1 | AF4/FMR2 family, member 1 [Source:HGNC Symbol;Acc:HGNC:7135] | 6 | -1.53 |
| NFAT5 | nuclear factor of activated T-cells 5, tonicity-responsive [Source:HGNC Symbol;Acc:HGNC:7774] | 18 | -1.53 |
| SH3BGRL | SH3 domain-binding glutamic acid-rich-like protein [Source:UniProtKB/Swiss-Prot;Acc:Q58DU7] | X | -1.53 |
| LRRC59 | Leucine-rich repeat-containing protein 59 [Source:UniProtKB/Swiss-Prot;Acc:Q5E9X4] | 19 | 1.53 |
| SLC28A3 | solute carrier family 28 member 3 [Source:RefSeq peptide;Acc:NP_001179096] | 8 | -1.53 |
| UNC5B | Bos taurus unc-5 homolog B (C. elegans) (UNC5B), mRNA. [Source:RefSeq mRNA;Acc:NM_001099029] | 28 | -1.53 |
| ABTB1 | ankyrin repeat and BTB/POZ domain-containing protein 1 [Source:RefSeq peptide;Acc:NP_001076853] | 22 | -1.53 |
| C12ORF2 | ras association domain-containing protein 8 [Source:RefSeq peptide;Acc:NP_001019720] | 5 | 1.53 |
| NSDHL | Sterol-4-alpha-carboxylate 3-dehydrogenase, decarboxylating [Source:UniProtKB/Swiss-Prot;Acc:Q3ZBE9] | X | -1.53 |
| PPFIA1 | liprin-alpha-1 [Source:RefSeq peptide;Acc:NP_001178306] | 29 | 1.53 |
| CALCOCO1 | calcium-binding and coiled-coil domain-containing protein 1 [Source:RefSeq peptide;Acc:NP_001039900] | 5 | -1.53 |
| MGC134574 | Bos taurus family with sequence similarity 221, member A (FAM221A), mRNA. [Source:RefSeq mRNA;Acc:NM_001075195] | 4 | -1.53 |
| USP19 | Ubiquitin carboxyl-terminal hydrolase [Source:UniProtKB/TrEMBL;Acc:F1MUD4] | 22 | -1.53 |
| PRSS22 | Uncharacterized protein [Source:UniProtKB/TrEMBL;Acc:E1BNJ9] | 25 | -1.53 |
| GATSL2 | Uncharacterized protein Source: UniProtKB/TrEMBL F1N308 | 25 | -1.53 |
| G3PDH | Glyceraldehyde-3-phosphate dehydrogenase [Source:UniProtKB/Swiss-Prot;Acc:P10096] | 5 | 1.53 |
| FABP5 | fatty acid binding protein 5 (psoriasis-associated) [Source:HGNC Symbol;Acc:HGNC:3560] | 14 | 1.53 |
| ZNF382 | zinc finger protein 382 [Source:RefSeq peptide;Acc:NP_001178367] | 18 | -1.53 |
| ACER3 | alkaline ceramidase 3 [Source:RefSeq peptide;Acc:NP_001095755] | 15 | 1.53 |
| C1S | complement C1s subcomponent precursor [Source:RefSeq peptide;Acc:NP_001070018] | 5 | 1.53 |
| NUDT11 | diphosphoinositol polyphosphate phosphohydrolase 3-alpha [Source:RefSeq peptide;Acc:NP_001030565] | X | 1.53 |
| DSP | desmoplakin [Source:RefSeq peptide;Acc:NP_001179297] | 23 | -1.53 |
| GRP78 | Uncharacterized protein [Source:UniProtKB/TrEMBL;Acc:F1N614] | 11 | -1.53 |
| FAM108C1 | Abhydrolase domain-containing protein FAM108C1 [Source:UniProtKB/Swiss-Prot;Acc:A5PKD9] | 21 | -1.53 |
| NAA50 | N-alpha-acetyltransferase 50 [Source:UniProtKB/Swiss-Prot;Acc:Q0IIJ0] | 1 | 1.53 |
| DOCK7 | dedicator of cytokinesis protein 7 [Source:RefSeq peptide;Acc:NP_001179835] | 3 | -1.53 |
| BRP44L | Mitochondrial pyruvate carrier 1 [Source:UniProtKB/Swiss-Prot;Acc:Q3ZCG2] | 9 | 1.53 |
| SEPT10 | septin-10 [Source:RefSeq peptide;Acc:NP_001039641] | 11 | -1.52 |
| LOC505468 | Uncharacterized protein [Source:UniProtKB/TrEMBL;Acc:F1N6N4] | 26 | -1.52 |
| DDX39 | ATP-dependent RNA helicase DDX39A [Source:RefSeq peptide;Acc:NP_001029924] | 7 | 1.52 |
| CD8B | T-cell surface glycoprotein CD8 beta chain precursor [Source:RefSeq peptide;Acc:NP_001098814] | 11 | -1.52 |
| SLC2A3 | solute carrier family 2, facilitated glucose transporter member 3 [Source:RefSeq peptide;Acc:NP_777028] | 5 | -1.52 |
| ARL8B | ADP-ribosylation factor-like protein 8B [Source:RefSeq peptide;Acc:NP_001039536] | 22 | 1.52 |
| ZFP36L1 | zinc finger protein 36, C3H1 type-like 1 [Source:RefSeq peptide;Acc:NP_001094704] | 10 | -1.52 |
| ACADS | short-chain specific acyl-CoA dehydrogenase, mitochondrial precursor [Source:RefSeq peptide;Acc:NP_001029573] | 17 | -1.52 |
| DYNC1LI2 | cytoplasmic dynein 1 light intermediate chain 2 [Source:RefSeq peptide;Acc:NP_001193081] | 18 | 1.52 |
| ZYG11B | protein zyg-11 homolog B [Source:RefSeq peptide;Acc:NP_001095717] | 3 | 1.52 |
| MOGAT1 | 2-acylglycerol O-acyltransferase 1 [Source:UniProtKB/Swiss-Prot;Acc:Q70VZ7] | 2 | 1.52 |
| ENSBTAG00000016074 | Uncharacterized protein [Source:UniProtKB/TrEMBL;Acc:F1N5N1] | 15 | 1.52 |
| GIPC2 | PDZ domain-containing protein GIPC2 [Source:UniProtKB/Swiss-Prot;Acc:Q1JQD4] | 3 | 1.52 |
| SRSF3 | Serine/arginine-rich splicing factor 3 [Source:UniProtKB/Swiss-Prot;Acc:Q3SZR8] | 23 | 1.52 |
| LINS | protein Lines homolog [Source:RefSeq peptide;Acc:NP_001192578] | 21 | 1.52 |
| FAM115C | protein FAM115C [Source:RefSeq peptide;Acc:NP_001095394] | 4 | 1.52 |
| ID1 | DNA-binding protein inhibitor ID-1 [Source:RefSeq peptide;Acc:NP_001091037] | 13 | -1.52 |
| CYCS | Cytochrome c [Source:UniProtKB/Swiss-Prot;Acc:P62894] | 4 | 1.52 |
| NUDT10 | nudix (nucleoside diphosphate linked moiety X)-type motif 10 | X | 1.52 |
| C10H5orf13 | neuronal protein 3.1 [Source:RefSeq peptide;Acc:NP_001098515] | 10 | -1.52 |
| HSPA14 | Heat shock 70 kDa protein 14 [Source:UniProtKB/Swiss-Prot;Acc:Q2YDD0] | 13 | 1.52 |
| RNF149 | Uncharacterized protein [Source:UniProtKB/TrEMBL;Acc:E1BHK5] | 11 | -1.52 |
| COMMD4 | COMM domain-containing protein 4 [Source:UniProtKB/Swiss-Prot;Acc:Q5E9V6] | 21 | 1.52 |
| Unknown 16 | unknown | 5 | -1.52 |
| FOXO4 | forkhead box protein O4 [Source:RefSeq peptide;Acc:NP_001094747] | X | -1.52 |
| ZNF569 | Uncharacterized protein [Source:UniProtKB/TrEMBL;Acc:F1MH55] | 18 | -1.52 |
| PPAT | amidophosphoribosyltransferase [Source:RefSeq peptide;Acc:NP_001095845] | 6 | 1.52 |
| SESN2 | Sestrin-2 [Source:UniProtKB/Swiss-Prot;Acc:Q58CN8] | 2 | 1.52 |
| ENSBTAG00000003197 | Novel processed pseudogene | 5 | 1.52 |
| DNAH10 | Uncharacterized protein [Source:UniProtKB/TrEMBL;Acc:F1MSP8] | 17 | -1.52 |
| TLN2 | Uncharacterized protein [Source:UniProtKB/TrEMBL;Acc:F1MQI1] | 10 | -1.52 |
| MRPL13 | 39S ribosomal protein L13, mitochondrial [Source:UniProtKB/Swiss-Prot;Acc:Q3SYS1] | 14 | 1.52 |
| OSBPL2 | oxysterol-binding protein-related protein 2 [Source:RefSeq peptide;Acc:NP_001030192] | 13 | -1.52 |
| BPTF | Uncharacterized protein [Source:UniProtKB/TrEMBL;Acc:F1N3U7] | 19 | -1.52 |
| INPP5K | inositol polyphosphate 5-phosphatase K [Source:RefSeq peptide;Acc:NP_001095456] | 19 | -1.52 |
| GXYLT1 | glucoside xylosyltransferase 1 [Source:HGNC Symbol;Acc:HGNC:27482] | 5 | 1.52 |
| RAB7B | Ras-related protein Rab-7b [Source:UniProtKB/Swiss-Prot;Acc:Q08DE8] | 16 | -1.52 |
| TSC22D1 | Bos taurus TSC22 domain family, member 1 (TSC22D1), mRNA. [Source:RefSeq mRNA;Acc:NM_001034377] | 12 | -1.52 |
| ENSBTAG00000031256 | Uncharacterized protein [Source:UniProtKB/TrEMBL;Acc:E1B773] | X | 1.52 |
| ESYT2 | Uncharacterized protein [Source:UniProtKB/TrEMBL;Acc:F1MN29] | 4 | -1.52 |
| PPIL5 | leucine-rich repeat protein 1 [Source:RefSeq peptide;Acc:NP_001098495] | 10 | 1.52 |
| S100A14 | Protein S100-A14 [Source:UniProtKB/Swiss-Prot;Acc:Q3MHP3] | 3 | 1.52 |
| PLSCR2 | Phospholipid scramblase 2 [Source:UniProtKB/Swiss-Prot;Acc:Q3ZBG9] | 1 | 1.52 |
| ARRDC2 | arrestin domain-containing protein 2 [Source:RefSeq peptide;Acc:NP_001075061] | 7 | 1.51 |
| Unknown 17 | unknown | 3 | -1.51 |
| PARP9 | poly [Source:RefSeq peptide;Acc:NP_001070296] | 1 | 1.51 |
| CKB | Creatine kinase B-type [Source:UniProtKB/Swiss-Prot;Acc:Q5EA61] | 21 | 1.51 |
| SLK | STE20-like kinase [Source:HGNC Symbol;Acc:HGNC:11088] | 26 | -1.51 |
| NUPR1 | nuclear protein 1 [Source:RefSeq peptide;Acc:NP_001107987] | 25 | 1.51 |
| RBP1 | Retinol-binding protein 1 [Source:UniProtKB/Swiss-Prot;Acc:P02694] | 1 | 1.51 |
| Unknown 18 | unknown | 19 | -1.51 |
| PQLC3 | PQ-loop repeat-containing protein 3 precursor [Source:RefSeq peptide;Acc:NP_001095348] | 11 | -1.51 |
| Unknown 18 | unknown | 13 | -1.51 |
| KLRC1 | KLRC1 protein; Uncharacterized protein [Source:UniProtKB/TrEMBL;Acc:A8E4Q0] | 5 | 1.51 |
| TUBB3 | Tubulin beta-3 chain [Source:UniProtKB/Swiss-Prot;Acc:Q2T9S0] | 18 | 1.51 |
| TMEM106A | Transmembrane protein 106A [Source:UniProtKB/Swiss-Prot;Acc:Q5EA90] | 19 | -1.51 |
| STX12 | syntaxin-12 [Source:RefSeq peptide;Acc:NP_001094523] | 2 | -1.51 |
| EXO1 | Uncharacterized protein [Source:UniProtKB/TrEMBL;Acc:E1BF15] | 16 | 1.51 |
| RNF113A | RING finger protein 113A [Source:UniProtKB/Swiss-Prot;Acc:Q67ER4] | X | 1.51 |
| C9orf91 | chromosome 9 open reading frame 91 [Source:HGNC Symbol;Acc:HGNC:24513] | 8 | -1.51 |
| MMS22L | Protein MMS22-like [Source:UniProtKB/Swiss-Prot;Acc:E1BGH8] | 9 | 1.51 |
| TMEM98 | transmembrane protein 98 [Source:RefSeq peptide;Acc:NP_001039611] | 19 | 1.51 |
| SHQ1 | Protein SHQ1 homolog [Source:UniProtKB/Swiss-Prot;Acc:Q3MHH1] | 22 | 1.51 |
| MCEE | Methylmalonyl-CoA epimerase, mitochondrial [Source:UniProtKB/Swiss-Prot;Acc:Q2KIZ3] | 21 | 1.51 |
| RGN | Regucalcin [Source:UniProtKB/Swiss-Prot;Acc:Q9TTJ5] | X | 1.51 |
| FARSB | phenylalanyl-tRNA synthetase beta chain [Source:RefSeq peptide;Acc:NP_001098491] | 2 | 1.51 |
| WAPAL | Uncharacterized protein [Source:UniProtKB/TrEMBL;Acc:E1BGC3] | 28 | 1.51 |
| LOC782608 | zinc finger protein 280B [Source:RefSeq peptide;Acc:NP_001071403] | 17 | 1.51 |
| SPRED1 | sprouty-related, EVH1 domain-containing protein 1 [Source:RefSeq peptide;Acc:NP_001179445] | 10 | -1.51 |
| SLCO4C1 | solute carrier organic anion transporter family member 4C1 [Source:RefSeq peptide;Acc:NP_001179775] | 7 | 1.51 |
| MAEL | protein maelstrom homolog [Source:RefSeq peptide;Acc:NP_001033282] | 3 | 1.51 |
| CBS | cystathionine beta-synthase [Source:RefSeq peptide;Acc:NP_001095470] | 1 | -1.51 |
| OCIAD2 | OCIA domain-containing protein 2 [Source:UniProtKB/Swiss-Prot;Acc:Q3SYY7] | 6 | 1.51 |
| HABP4 | intracellular hyaluronan-binding protein 4 [Source:RefSeq peptide;Acc:NP_001074992] | 8 | -1.51 |
| ABCB1 | Uncharacterized protein [Source:UniProtKB/TrEMBL;Acc:G3X771] | 4 | -1.51 |
| PRUNE2 | PRUNE2 protein; Uncharacterized protein [Source:UniProtKB/TrEMBL;Acc:A7Z048] | 8 | -1.51 |
| SYPL1 | synaptophysin-like protein 1 [Source:RefSeq peptide;Acc:NP_001103558] | 4 | 1.51 |
| BAG3 | BAG family molecular chaperone regulator 3 [Source:RefSeq peptide;Acc:NP_001075940] | 26 | 1.51 |
| PRICKLE1 | prickle-like protein 1 [Source:RefSeq peptide;Acc:NP_001096004] | 5 | -1.50 |
| DNAJA1 | DnaJ homolog subfamily A member 1 [Source:UniProtKB/Swiss-Prot;Acc:Q5E954] | 8 | 1.50 |
| TBCE | Tubulin-specific chaperone E [Source:UniProtKB/Swiss-Prot;Acc:Q32KS0] | 28 | -1.50 |
| ENSBTAG00000021026 | Known pseudogene | X | 1.50 |
| KIAA0528 | uncharacterized protein KIAA0528 homolog [Source:RefSeq peptide;Acc:NP_001095973] | 5 | -1.50 |
| HTATIP2 | oxidoreductase HTATIP2 [Source:RefSeq peptide;Acc:NP_001035653] | 29 | 1.50 |
| IL4R | interleukin-4 receptor subunit alpha precursor [Source:RefSeq peptide;Acc:NP_001068610] | 25 | -1.50 |
| TPM4 | tropomyosin alpha-4 chain [Source:RefSeq peptide;Acc:NP_001094632] | 7 | 1.50 |
| LFNG | Beta-1,3-N-acetylglucosaminyltransferase lunatic fringe [Source:UniProtKB/Swiss-Prot;Acc:Q2KJ92] | 25 | -1.50 |
| ANXA1 | annexin A1 [Source:RefSeq peptide;Acc:NP_786978] | 8 | -1.50 |
| CCDC109B | coiled-coil domain-containing protein 109B [Source:RefSeq peptide;Acc:NP_001068639] | 6 | -1.50 |
| FTH1 | Ferritin heavy chain [Source:UniProtKB/Swiss-Prot;Acc:O46414] | 29 | 1.50 |
| MRPL18 | 39S ribosomal protein L18, mitochondrial [Source:UniProtKB/Swiss-Prot;Acc:Q3ZBR7] | 9 | 1.50 |

All genes differentially expressed (1.5‑fold; p<0.05) according to microarray data are listed with their chromosome. Negative fold change (FC) values indicate lower expression levels in NHSM ICMs and positive values are at higher expression levels in NHSM ICMs compared with SOF ICMs.
